# Supplementary material for: Comparative study of Taqman-based qPCR assay for the detection of Anisakis simplex and Pseudoterranova decipiens
Source: PLoS One. 2025 Apr 25;20(4):e0320724. doi: 10.1371/journal.pone.0320724 (PMC12027023; doi:10.1371/journal.pone.0320724)
Supplement: S1 Table — From the list, select only those that are applicable to this paper. All essential information (E) must be submitted with the manuscript. Desirable information (D) should be submitted if available. If using primers obtained from RTPrimerDB, information on qPCR target, oligonucleotides, protocols and validation is available from that source. FFPE, formalin-fixed, paraffin-embedded; RIN, RNA integrity number; RQI, RNA quality indicator; GSP, gene-specific priming; dNTP, deoxynucleoside triphosphate. (DOCX) [file pone.0320724.s001.docx]

| **ITEM TO CHECK** | **IMPORTANCE** | **ITEM TO CHECK** | **IMPORTANCE** |
| --- | --- | --- | --- |
| **EXPERIMENTAL DESIGN** | | **qPCR PROTOCOL** | |
| Definition of experimental and control groups | E | Complete reaction conditions | E |
| Number within each group | E | Reaction volume and amount of cDNA/DNA | E |
| Assay carried out by core lab or investigator's lab? | D | Primer, (probe), Mg++ and dNTP concentrations | E |
| Acknowledgement of authors' contributions | D | Polymerase identity and concentration | E |
| **SAMPLE** | | Buffer/kit identity and manufacturer | E |
| Description | E | Additives (SYBR Green I, DMSO, etc.) | E |
| Microdissection or macrodissection | E | Complete thermocycling parameters | E |
| Processing procedure | E | Reaction setup (manual/robotic) | D |
| If fixed - with what, how quickly? | E | Manufacturer of qPCR instrument | E |
| Sample storage conditions and duration (especially for FFPE samples) | E | **qPCR VALIDATION** | |
| **NUCLEIC ACID EXTRACTION** | | Evidence of optimization (from gradients) | D |
| Procedure and/or instrumentation | E | Specificity (gel, sequence, melt, or digest) | E |
| Name of kit and details of any modifications | E | Standard curves with slope and y-intercept | E |
| Details of DNase or RNAse treatment | E | PCR efficiency calculated from slope | E |
| Contamination assessment (DNA or RNA) | E | r^2^ of standard curve | E |
| Nucleic acid quantification | E | C_q_ variation at lower limit | E |
| Instrument and method | E | Evidence for limit of detection | E |
| Inhibition testing (C_q_ dilutions, spike or other) | E | If multiplex, efficiency and LOD of each assay. | E |
| **qPCR TARGET INFORMATION** | | **DATA ANALYSIS** | |
| If multiplex, efficiency and LOD of each assay. | E | qPCR analysis program (source, version) | E |
| Sequence accession number | E | Cq method determination | E |
| Location of amplicon | D | Outlier identification and disposition | E |
| Amplicon length | E | Results of NTCs | E |
| *In silico* specificity screen (BLAST, etc.) | E | Justification of number and choice of reference genes | E |
| Location of each primer by exon or intron (if applicable) | E | Description of normalization method | E |
| What splice variants are targeted? | E | Number and concordance of biological replicates | D |
| **qPCR OLIGONUCLEOTIDES** | | Number and stage (RT or qPCR) of technical replicates | E |
| Primer sequences | E | Repeatability (intra-assay variation) | E |
| Probe sequences | D* | Reproducibility (inter-assay variation, %CV) | D |
| Location and identity of any modifications | E | Software (source, version) | E |
| Manufacturer of oligonucleotides | D |  |  |

*: Disclosure of the probe sequence is highly desirable and strongly encouraged. However, since not all commercial pre-designed assay vendors provide this information, it cannot be an essential requirement. Use of such assays is advised against.

**S1 Table** Partial list of MIQE checklists [1]. From the list, select only those that are applicable to this paper. All essential information (E) must be submitted with the manuscript. Desirable information (D) should be submitted if available. If using primers obtained from RTPrimerDB, information on qPCR target, oligonucleotides, protocols and validation is available from that source. FFPE, formalin-fixed, paraffin-embedded; RIN, RNA integrity number; RQI, RNA quality indicator; GSP, gene-specific priming; dNTP, deoxynucleoside triphosphate.

**Supporting references**

1. Bustin SA, Benes V, Garson JA, Hellemans J, Huggett J, Kubista M, et al. The MIQE guidelines: minimum information for publication of quantitative real-time PCR experiments. Clin Chem. 2009;55(4):611–22. Available from: http://dx.doi.org/10.1373/clinchem.2008.112797
