# Supplementary material for: Comparative study of Taqman-based qPCR assay for the detection of Anisakis simplex and Pseudoterranova decipiens
Source: PLoS One. 2025 Apr 25;20(4):e0320724. doi: 10.1371/journal.pone.0320724 (PMC12027023; doi:10.1371/journal.pone.0320724)
Supplement: Attachment — Submitted filename: PONE-D-24-33370.pdf [file pone.0320724.s002.pdf]

PLOS ONE

Comparative study of Taqman-based real-time PCR assay for the detection of Anisakis simplex and Pseudoterranova decipiens

--Manuscript Draft--

**Manuscript Number:**	PONE-D-24-33370
**Article Type:**	Research Article
**Full Title:**	Comparative study of Taqman-based real-time PCR assay for the detection of Anisakis simplex and Pseudoterranova decipiens
**Short Title:**	qPCR assay for Anisakid detection
**Corresponding Author:**	Migyeong Kim<br>Ministry of Food and Drug Safety<br>Heungdeok-gu, Cheongju-si, KOREA, REPUBLIC OF
**Keywords:**	Anisakis, qPCR, intestinal parasitic infection, Anisakis simplex, Pseudoterranova decipiens
**Abstract:**	Anisakiasis is a foodborne parasitic infection caused by the consumption of raw or uncooked seafood that contains third-instar larvae from the Anisakidae family. This infection occurs worldwide but is particularly evident in South Korea and Japan. Therefore, it is necessary to compare and analyze the optimal detection methods to prevent Anisakis outbreaks. Therefore, we aimed to develop a species-specific Taqman-based real-time PCR method for detecting the internal transcribed spacer region and mtDNA genes of Anisakis simplex and Pseudoterranova decipiens. Parasite-specific primer/probe sets were selected based on the data from domestic and foreign detection methods. Additionally, we designed our own primer/probe sets based on the target region of each parasite. Through a literature review and self-creation process, seven detection method sets of A. simplex and P. decipiens were selected, and their sensitivity was checked by comparing the Ct values from the extracted DNA. The concentrations of six primer/probe sets detected through the screening process were compared to optimize the test method. The optimized method showed a limit of detection of 0.0019 ng/μL for A. simplex and 0.0001 ng/μL for P. decipiens. The specificity test also confirmed that there was no cross-activity with the five parasite samples and the three types of anisakids plasmid DNA. This study would contribute to the rapid detection method for Anisakis and provide a foundation for proactive response of food poisoning outbreaks.
**Order of Authors:**	Migyeong Kim
	Min Ji Hong
	Doo Won Seo
	Hyun Mi Jung
	Hyun-Ja Han
	Seung Hwan Kim
	Insun Joo
**Additional Information:**	
**Question**	**Response**
**Financial Disclosure**<br><br>Enter a financial disclosure statement that describes the sources of funding for the work included in this submission. Review the submission guidelines for detailed requirements. View published research articles from *PLOS ONE* for specific	Yes

Powered by Editorial Manager® and ProduXion Manager® from Aries Systems Corporation

examples.<br><br>This statement is required for submission and **will appear in the published article** if the submission is accepted. Please make sure it is accurate.<br><br>**Funded studies**<br>Enter a statement with the following details:<br>• Initials of the authors who received each award<br>• Grant numbers awarded to each author<br>• The full name of each funder<br>• URL of each funder website<br>• Did the sponsors or funders play any role in the study design, data collection and analysis, decision to publish, or preparation of the manuscript?<br><br>Did you receive funding for this work?	
Please add funding details.<br>as follow-up to "**Financial Disclosure**<br><br>Enter a financial disclosure statement that describes the sources of funding for the work included in this submission. Review the submission guidelines for detailed requirements. View published research articles from *PLOS ONE* for specific examples.<br><br>This statement is required for submission and **will appear in the published article** if the submission is accepted. Please make sure it is accurate.<br><br>**Funded studies**<br>Enter a statement with the following details:<br>• Initials of the authors who received each award<br>• Grant numbers awarded to each author<br>• The full name of each funder<br>• URL of each funder website<br>• Did the sponsors or funders play any role in the study design, data collection and analysis, decision to publish, or preparation of the manuscript?	This study was supported by the Ministry of Food and Drug Safety as part of the Ministry's R&D project.

Powered by Editorial Manager® and ProduXion Manager® from Aries Systems Corporation

Did you receive funding for this work?"	
Please select the country of your main research funder (please select carefully as in some cases this is used in fee calculation).<br>as follow-up to "**Financial Disclosure**<br><br>Enter a financial disclosure statement that describes the sources of funding for the work included in this submission. Review the submission guidelines for detailed requirements. View published research articles from *PLOS ONE* for specific examples.<br><br>This statement is required for submission and **will appear in the published article** if the submission is accepted. Please make sure it is accurate.<br><br>**Funded studies**<br>Enter a statement with the following details:<br>• Initials of the authors who received each award<br>• Grant numbers awarded to each author<br>• The full name of each funder<br>• URL of each funder website<br>• Did the sponsors or funders play any role in the study design, data collection and analysis, decision to publish, or preparation of the manuscript?<br><br>Did you receive funding for this work?"	KOREA, REPUBLIC OF - KR
**Competing Interests**<br><br>Use the instructions below to enter a competing interest statement for this submission. On behalf of all authors, disclose any competing interests that could be perceived to bias this work—acknowledging all financial support and any other relevant financial or non-financial competing interests.	The authors have declared that no competing interests exist.

Powered by Editorial Manager® and ProduXion Manager® from Aries Systems Corporation

This statement is required for submission and **will appear in the published article** if the submission is accepted. Please make sure it is accurate and that any funding sources listed in your Funding Information later in the submission form are also declared in your Financial Disclosure statement.<br><br>View published research articles from *PLOS ONE* for specific examples.<br><br>**NO authors have competing interests**<br><br>Enter: *The authors have declared that no competing interests exist.*<br><br>**Authors with competing interests**<br><br>Enter competing interest details beginning with this statement:<br><br>*I have read the journal's policy and the authors of this manuscript have the following competing interests: [insert competing interests here]*<br><br>* typeset	
**Ethics Statement**<br><br>Enter an ethics statement for this submission. This statement is required if the study involved:<br><br>• Human participants<br>• Human specimens or tissue<br>• Vertebrate animals or cephalopods<br>• Vertebrate embryos or tissues<br>• Field research<br><br>Write "N/A" if the submission does not require an ethics statement.<br><br>General guidance is provided below. Consult the submission guidelines for detailed instructions. **Make sure that all information entered here is included in the Methods section of the manuscript.**	N/A

Powered by Editorial Manager® and ProduXion Manager® from Aries Systems Corporation

**Format for specific study types**<br><br>**Human Subject Research (involving human participants and/or tissue)**<br>• Give the name of the institutional review board or ethics committee that approved the study<br>• Include the approval number and/or a statement indicating approval of this research<br>• Indicate the form of consent obtained (written/oral) or the reason that consent was not obtained (e.g. the data were analyzed anonymously)<br><br>**Animal Research (involving vertebrate animals, embryos or tissues)**<br>• Provide the name of the Institutional Animal Care and Use Committee (IACUC) or other relevant ethics board that reviewed the study protocol, and indicate whether they approved this research or granted a formal waiver of ethical approval<br>• Include an approval number if one was obtained<br>• If the study involved *non-human primates*, add *additional details* about animal welfare and steps taken to ameliorate suffering<br>• If anesthesia, euthanasia, or any kind of animal sacrifice is part of the study, include briefly which substances and/or methods were applied<br><br>**Field Research**<br><br>Include the following details if this study involves the collection of plant, animal, or other materials from a natural setting:<br>• Field permit number<br>• Name of the institution or relevant body that granted permission	
**Data Availability**<br><br>Authors are required to make all data underlying the findings described fully available, without restriction, and from the time of publication. PLOS allows rare exceptions to address legal and ethical concerns. See the PLOS Data Policy and FAQ for detailed information.	Yes - all data are fully available without restriction

Powered by Editorial Manager® and ProduXion Manager® from Aries Systems Corporation

A Data Availability Statement describing where the data can be found is required at submission. Your answers to this question constitute the Data Availability Statement and **will be published in the article**, if accepted.<br><br>**Important:** Stating 'data available on request from the author' is not sufficient. If your data are only available upon request, select 'No' for the first question and explain your exceptional situation in the text box.<br><br>Do the authors confirm that all data underlying the findings described in their manuscript are fully available without restriction?	
**Describe where the data may be found in full sentences. If you are copying our sample text, replace any instances of XXX with the appropriate details.**<br><br>• If the data are **held or will be held in a public repository**, include URLs, accession numbers or DOIs. If this information will only be available after acceptance, indicate this by ticking the box below. For example: *All XXX files are available from the XXX database (accession number(s) XXX, XXX.).*<br>• If the data are all contained **within the manuscript and/or Supporting Information files**, enter the following: *All relevant data are within the manuscript and its Supporting Information files.*<br>• If neither of these applies but you are able to provide **details of access elsewhere**, with or without limitations, please do so. For example:<br><br>*Data cannot be shared publicly because of [XXX]. Data are available from the XXX Institutional Data Access / Ethics Committee (contact via XXX) for researchers who meet the criteria for access to confidential data.*<br><br>*The data underlying the results presented in the study are available*	ll relevant data are within the manuscript and its Supporting Information files.

from (include the name of the third party and contact information or URL).<br>• This text is appropriate if the data are owned by a third party and authors do not have permission to share the data.<br><br>* typeset	
Additional data availability information:	Tick here if the URLs/accession numbers/DOIs will be available only after acceptance of the manuscript for publication so that we can ensure their inclusion before publication.

Powered by Editorial Manager® and ProduXion Manager® from Aries Systems Corporation

Manuscript Click here to access/download;Manuscript;(Manuscript).docx

Comparative study of Taqman-based real-time PCR assay for the detection of *Anisakis simplex* and *Pseudoterranova decipiens*

Short title: qPCR assay for Anisakids detection

Mi-Gyeong Kim[1]*, Min Ji Hong [1], Doo Won Seo[1]**, Hyun Mi Jung[2], Hyun-Ja Han[1], Seung Hwan Kim[1], Insun Joo[1]

Affiliations

[1] Food Microbiology Division, National Institute of Food and Drug Safety Evaluation, Ministry of Food and Drug Safety, 187 Osongsaengmyeong 2-ro, Osong-eup, Heungdeok-gu, Cheongju-si, Chungcheongbuk-do, 28159, Republic of Korea

[2] National Institute of Fisheries Science, 14, Seonnyeobawi-ro, Jung-gu, Incheon, Republic of Korea

Abbreviation

mtDNA: mitochondrial DNA

ITS: Internal transcribed spacer

qPCR: Real-time polymerase chain reaction

Corresponding author

* Mi-Gyeong Kim: kmg129@korea.kr

** Doo Won Seo: realize17@korea.kr

Phone number: 82-43-719-4305 Fax number: 82-43-719-4300

Author contributions

Mi-Gyeong Kim: Investigation, Writing-Original Draft Preparation, kmg129@korea.kr

Min Ji Hong: Investigation, Writing-Original Draft Preparation, congmj90@korea.kr

Doo Won Seo: Methodology, Writing-Review & Editing, realize17@korea.kr

Hyun Mi Jung: Supervision, Writing-Review & Editing, jeonghm@korea.kr

Hyun-Ja Han: Supervision, Writing-Review & Editing, hjhan77@korea.kr

Seung Hwan Kim: Supervision, betterkim@korea.kr

Insun Joo: Supervision, jis901@korea.kr

Abstract

Anisakiasis is a foodborne parasitic infection caused by the consumption of raw or uncooked seafood that contains third-instar larvae from the Anisakidae family. This infection occurs worldwide but is particularly evident in South Korea and Japan. Therefore, it is necessary to compare and analyze the optimal detection methods to prevent Anisakis outbreaks. Therefore, we aimed to develop a species-specific Taqman-based real-time PCR method for detecting the internal transcribed spacer region and mtDNA genes of *Anisakis simplex* and *Pseudoterranova decipiens*. Parasite-specific primer/probe sets were selected based on the data from domestic and foreign detection methods. Additionally, we designed our own primer/probe sets based on the target region of each parasite. Through a literature review and self-creation process, seven detection method sets of *A. simplex* and *P. decipiens* were selected, and their sensitivity was checked by comparing the Ct values from the extracted DNA. The concentrations of six primer/probe sets detected through the screening process were compared to optimize the test method. The optimized method showed a limit of detection of 0.0019 ng/μL for *A. simplex* and 0.0001 ng/μL for *P. decipiens*. The specificity test also confirmed that there was no cross-activity with the five parasite samples and the three types of anisakids plasmid DNA. This study would contribute to the rapid detection method for Anisakis and provide a foundation for proactive response of food poisoning outbreaks.

Keywords: Anisakis, qPCR, intestinal parasitic infection, *Anisakis simplex*, *Pseudoterranova decipiens*

Introduction

Anisakiasis, a zoonotic disease that occurs worldwide, is caused by the larvae of nematodes that parasitize the stomachs of marine mammals, such as whales, dolphins, and seals. Anisakiasis is a parasitic infectious disease transmitted to humans by consuming infected raw fish ~~infected~~ [1].

Anisakis lay thousands of eggs in the whale's body, which are then shed in feces. After floating in water for a while, the larvae develop into the third larval stage (L3) in crustaceans of fish that have consumed crustaceans. These crustaceans are then predated upon by fish or squid, where the larvae mature into adults in the intestines. These L3-infected fish or cephalopods are then ingested raw and infect humans, causing gastrointestinal illnesses [2]. Anisakiasis refers to infection with roundworms and is accompanied by symptoms, such as vomiting, chills, and intense pain. IgE-mediated allergic reactions have also been reported [3,4].

Anisakis is widely distributed worldwide, including oceans, with Korea being a key endemic region, along with Japan, the Netherlands, Spain, and Germany. In particular, Japan has experienced a continuous outbreak of whale roundworm disease caused mainly by *Anisakis simplex* and *A. pegreffi* because of the frequent consumption of raw fish [5–7]. In Korea, cases of infection have been consistently reported; thus, its global prevalence can be confirmed [8–10].

The family Anisakidae consists of the genera *Anisakis*, *Pseudoterranova,* and *Contracaecum*, and the larvae can be morphologically classified according to the Anisakis species [11]. The most commonly infected species is the Anisakis genus, followed by *Pseudoterranova decipiens*, and rarely *Contracaecum* and *Hysterothylacium* [12]. Recently, *A. simplex* was reported to

increase the prevalence of hypersensitivity in freezing fish [13]. Meanwhile, the infection rate of *P. decipiens* is correlated with an increase in the number of marine mammals in this area [14].

Based on this, molecular detection methods that can be appropriately used for the detection of *A. simplex* and *P. decipiens* in seafood are needed. In this study, we aimed to develop a species-specific TaqMan-based quantitative-polymerase chain reaction (qPCR) method for detecting mtDNA and ITS regions, as well as validate each parasite test method to urgently address food poisoning incidents, such as anisakiasis.

Materials and methods

DNA extraction

Anisakis larvae isolated from marine mammals were provided by the Cetacean Research Institute, National Institute of Fisheries Science, and stored in 100% ethanol until DNA extraction. DNA extraction was performed using the DNeasy Blood & Tissue kit (Qiagen) following the steps for the Purification of Total DNA from Animal tissues (spin column protocol). DNA was extracted by cutting a 1-cm section of the body of a single Anisakis larva, and DNA quality was checked at a 260/280 nm ratio using a Nanodrop (Thermo Fisher Scientific, USA). DNA extracts were stored at -20 °C until use.

Design of primers and probe

Before constructing the primer and probe sets for Anisakis detection, we collected and analyzed research data on domestic and foreign parasite gene detection methods and cataloged the primers/probes for the parasite. Based on the unique target regions that could be identified, we focused on a qPCR detection method using TaqMan probes. Among the initially selected primer/probe sets, the detection region was checked using BLAST (https://blast.ncbi.nlm.nih.gov/Blast.cgi), and sets that were not cross-detected were secondarily selected.

In addition to those obtained from the literature, we designed primers/probes for verification. The sequences of *A. simplex* and *P. decipiens* mtDNA cox2 obtained from the National Center for Biotechnology Information were aligned using the Mega 11 program. The most common sequence resulting from the alignment was used as the template sequence (*A. simplex*

(LC543759), *P. decipiens* (AB576757.1)) and subjected to Genscript (optimal primer size: 20 bp, optimal primer Tm: 60.0 °C, optimal product size: 200 bp), and IDT (primer size: 17–30 bp, primer Tm: 59-65 °C, primer GC content: 35–65%, product size: 75–150 bp) using the homepage services of Genscript (optimal primer size: 20 bp, optimal primer Tm: 60). SnapGene™ 1.1.3 program was used to check the position of the primer/probe set to the template sequence. All the primers were synthesized by Genotech (Daejeon, Korea) and all probes were synthesized by Bioneer (Daejeon, Korea).

qPCR optimization

The real-time PCR mixtures for each method were Taqman Universal Master mix II (Applied Biosystems, USA), which was configured to the following concentrations: 2 µL of DNA (*A. simplex*: 7.4 ng/µL, *P. decipiens*: 10.7 ng/µL), 10 µL of Universal mater mix, 400 and 500 nM of primers, and 200 and 250 nM of probe, and distilled water was added to attain a total volume of 20 µL.

PCR amplification was performed using the 7500 Real-Time PCR System (Applied Biosystems). The PCR conditions were adapted from the respective reference methods. In-house PCR conditions were as follows: cDNA synthesis at 50 °C for 2 min, pre-heating at 95 °C for 10 min, denaturation at 95 °C for 15 s for 40 cycles, and annealing and extension at 57–61 °C for 1 min. Each real-time PCR sample was analyzed in triplicate.

Specificity and limit of detection (LOD) analysis

To verify the specificity of the finalized *A. simplex* and *P. decipiens* gene detection test methods, nine other species parasites and two larvae were used to confirm non-specific detection. Samples positive for the nine species parasites were collected from the Parasite

Laboratory of the Korea Association of Health Promotion. Nucleic acids of the five identified parasites (*Clonorchis sinensis*, *Toxoplasma gondii*, *Metagonimus yokogawai*, *Gymnophalodides seoi,* and *Taenia solium*) were extracted. Plasmid DNA from *Anisakis physeteris*, *Contracaecum osculatum*, and *Pseudoterranova azarasi* were used as templates. The specificity of the selected samples was verified using real-time PCR.

To compare the LOD with the selected primers and probe sets, *A. simplex* and *P. decipiens* DNA products underwent 1/10-fold stepwise dilution and were analyzed across a concentration range of 10^0–10^{10} copies/μL.

Synthesis of positive control

Positive controls were designed for the target sites of the final *A. simplex* and *P. decipiens* gene detection methods. A portion of the template genome sequence, including the detection site considered in the primer design for each parasite, was used as the positive control. The positive control gene was synthesized by Bioneer (Daejeon, Korea).

Inter-examinator validation

Inter-examiner validation of the method was performed by two investigators in our laboratory once daily for a total of 3 d. The DNA extracted from the anisakids was diluted 10^{-3}-fold (1xLOD) and 5x 10^{-3}-fold (5xLOD) for *A. simplex* and 10^{-5}-fold (1xLOD) and 10^{-5}-fold (5xLOD) for *P. decipiens*. Real-time PCR was then performed using the diluted DNA as templates together with the final detection method selected for this study. All experiments were performed in triplicate.

Results and Discussion

A literature search of the last 10 years for Taqman-based real-time PCR gene detection in anisakid (Table 1) revealed six studies concerning *A. simplex* and one for *P. decipiens* [2, 16-20]. In addition, primer/probe sets were designed based on the sequences of *A. simplex* (LC543759) and *P. decipiens* (AB576757.1): one for *A. simplex* and five for *P. decipiens* (Table 2). In total, seven detection methods were selected for the comparison between *A. simplex* and *P. decipiens* (Table 1, 2). The detection regions of the selected primer/probe sets were first checked for overlap with those of other Anisakis species and genera using BLAST, and their specificity was confirmed via real-time PCR using actual parasite samples from various species (Fig. 1). As a result, *A. simplex* and *P. decipiens* each showed one independent amplification curve, confirming that there was no cross-reaction between species.

Fig. 1 Specificity for optimized and Real-time PCR methods to detect anisakids. The amplification curves of ascaridoid species were verified using the other 9 parasites. (A) *A. simplex* (B) *P. decipiens*

Table 1 Selected sets of primers and probes for detection based on reference

Type	Primer name	Target	Sequence (5′-3′)[a]	Product size (bp)	Reference
A. simplex complex	AS1_F	ITS	GACTGTGAAGCATTCGGCAAG	161	[21]
	AS1_R		ACCACCAAAAGCACCTCACC		
	AS1_P		FAM-CTATCATGGACAATATGACGAGCGGTTC-TAMRA		
	ASs1_F		TTTTGGCTGCTAATCATCATTGA	76	[15]
	ASs1_R		CCACCTAGCGTGGCTCATTAA		
	ASs1_P		VIC-TAGCTTAAGGCAGAGTTG-MGB		
	ASs5_F	ITS1	GAACAACGGTGACCAATTTGG	56	[16]
	ASs5_R		GACGGTCCAGGCAGAAGCT		
	ASs5_P		VIC-TACGCCGTATCTAGCTTC-MGB		
	ASs2_F	COX2	CTTTAATTTTGGTTGCTCAGAT	217	[17]
	ASs2_R		CGATTATCAACCTCCAAAAG		
	ASs2_P		CY-ATGACCAGTGACTTTCACAGTCAAAT-BBQ		
	ASs3_F		AGTAAGAAGATTGAATATCAGTTTGGTGA	61	[18]
	ASs3_R		AAGTAAACTCAAAGAAGGCACCATC		
	ASs3_P		FAM-TTCCTACTTTAATTTTGGTTGCTC-MGB		
A. simplex complex & P. decipiens	AS2_F	COX1	GGKCYATTAAYTYTATRACWACTAC	176	[19]
	AS2_R		AAAGAWGTATTMARRTTACGRTCVG		
	AS2_P		FAM-TCTATTTCTTTGGARCAYA-TAMRA		

[a] Mixed based; R = A + G, Y = C + T, S = G + C, W = A + T, K = G + T, M = A + C, B = C + G + T, D = A + G + T, H = A + C + T, V = A + C + G, N = any base

Table 2 Selected sets of newly designed primers and probes for the detection of *A. simplex* and *P. decipiens*

Type	Primer name	Target	Sequence (5′-3′)	Product size (bp)
A. simplex	ASS pair1_F		CCGGGCTTAGAGTTTGA	103
complex	ASS pair1_R		CACAAGGGACAACACAACGA	
	ASS pair1_P		FAM-ACGAGGCTCGCCTAGCTCCA-TAMRA	
P. decipiens	PD pair1_F		AGAGACATTCCTGGTTTGGAGT	172
	PD pair1_R		CTAGGCAAAGCCCAAGAA	
	PD pair1_P		FAM-ACGTGGCTCTCCCAATTCCAACTGA-TAMRA	
	PD pair1 J_F		TCCTGGTTTGGAGTTTGATTCT	101
	PD pair1 J_R		CACAAGGAACAACACAACGATTA	
	PD pair1 J_P		FAM-TCAACCTCTAACAAACGTGGCTCCC-TAMRA	
	PD pair2 J_F		CGTTGTGTTGTTCCTTGTGATAC	111
	PD pair2 J_R	COX2	ACCTCTCATAGCATCCAACTTAAT	
	PD pair2 J_P		FAM-TTCATTCTTGAGCTTTGCCCAGGA -TAMRA	
	PD pair3 J_F		GGGAGCCACGTTTGTTAGA	107
	PD pair3 J_R		GGGCAAAGCTCAAGAATGAATTA	
	PD pair3 J_P		FAM-TCACAAGGAACAACACAACGATTATCAACC-TAMRA	
	PD pair4 J_F		GCCACGTTTGTTAGAGGTTGATA	106
	PD pair4 J_R		CCTGGGCAAAGCTCAAGAA	
	PD pair4 J_P		FAM-TCGTTGTGTTGTTCCTTGTGATACT-TAMRA	
	PD pair5 J_F		GGGATATTCCTGGTTTGGAGTT	121
	PD pair5 J_R		CGAATATTAGTATCACAAGGAACAACAC	
	PD pair5 J_P		FAM-TCAACCTCTAACAAACGTGGCTCCC-TAMRA	

Sensitivity of primers and probes for the identification of anisakids and qPCR setup

Comparative experiments were conducted to confirm the detection of Anisakis using the selected reference and constructed test methods. The real-time PCR conditions used the PCR conditions of the selected reference and the parasite gene detection method in the Guidance Documents of Food Poisoning Test [21] (Table 3). The results showed that three instances of *A. simplex* and five instances of *P. decipiens* were detected. Therefore, we selected each primer/probe set that confirmed the detection of Anisakis during the test according to the primer set.

Using the reference concentration (400 nM:200 nM) listed in the Guidance Documents of Food Poisoning [21], the selected sets were compared using different primer and probe concentrations. The method with the most stable Ct value (< 30) was selected based on a comparison between methods using actual extracted DNA [16] (Table 3). The primer:probe concentration combinations (400 nM:200 nM, 400 nM:250 nM, 500 nM:200 nM, and 500 nM:250 nM) were applied as PCR conditions for each set. We further optimized the detection method by analyzing qPCR assays with different primer/probe set concentration combinations. The final concentrations selected were 400 nM/250 nM for *A. simplex* and 400 nM/200 nM for *P. decipiens* (Table 4).

Table 3 Screening of primer/probe sets for each anisakid sample

Type	Region	Primer	Product size (bp)	Ct value
A. simplex	ITS	AS1	161	ND
		ASS1	76	ND
	ITS1	ASS5	56	38.53
	COI	AS2	178	ND
	COX2	ASS2	217	ND
		ASS3	96	23.77
		ASS pair1	103	39.79
P. decipiens	COI	AS2	178	ND
	COX2	PD pair1	172	ND
		PD pair1 J	101	19.77
		PD pair2 J	111	24.52
		PD pair3 J	107	21.75
		PD pair4 J	106	22.76
		PD pair5 J	121	20.62

ND, Not detected

Table 4 Combination of primers and probes with different concentration for comparison of Ct values using real-time PCR

Type	Primer	Primers:Probe (nM:nM)			
		400:200	400:250	500:200	500:250
A. simplex	ASS3	22.643±0.139	22.421±0.062	22.433±0.122	22.499±0.162
P. decipiens	PD pair1 J	20.496±0.024	23.543±0.25	22.735±1.322	23.181±0.084
	PD pair2 J	22.674±1.603	23.463±0.125	23.218±0.299	23.302±0.202
	PD pair3 J	24.204±1.258	22.799±0.095	22.269±0.716	23.143±0.026
	PD pair4 J	22.128±0.253	22.254±0.154	21.789±0.209	21.147±0.381
	PD pair5 J	21.626±0.091	21.911±0.172	22.109±0.108	22.846±0.179

The data are represented as mean±standard deviation (SD)

Specificity of optimal qPCR method

Specificity validation revealed that both *A. simplex* and *P. decipiens* were specifically detected without cross-validation. Gene amplification was not detected in parasites of other species or anisakids of the same species. The real-time PCR amplification curves showed that only the corresponding anisakids were amplified (Fig. 1). This confirmed that the selected detection method was valid for the specific detection of certain anisakids.

LOD

The extracted DNA was diluted 1/10-fold stepwise and used as a template. The detection limits were confirmed as 1.93×10^7 copies/µL for *A. simplex* and 9.67×10^5 copies/µL for *P. decipiens*. In addition, the amplification efficiency (E) was 91% for *A. simplex* and 110% for *P. decipiens*, and their corresponding correlation coefficients (R^2) were 0.9998 and 0.9949, respectively (Fig. 2). This strong linear relationship (R^2>0.99) indicates that the qPCR assay is suitable for Anisakis detection.

Based on the optimized test method, the LODs were 0.0019 and 0.0001 ng, with high sensitivity for *A. simplex and P. decipiens*, respectively. This obtained sensitivity is consistent with other studies [16,17,19,28], where LODs ranged from 0.3 to 2 pg, with *P. decipiens* detected down to 0.1 pg. Additionally, Ct values were also within acceptable tolerances in cross-validation.

Fig. 2 Standard curve of the Ct value and logarithm of *A. simplex* and *P. decipiens* cell numbers. Linearity test of 10-fold diluted series of (A) *A. simplex* and (B) *P. decipiens* DNA samples

Cross-examiner validation

To ensure the reliability of the anisakids gene test method selected in this study, cross-validation was conducted in triplicate between the two investigators. As a result of detection by real-time PCR, the CV values of 1 x LOD and 5 x LOD of *A. simplex* gene amplification products of Investigator A were 0.50% and 0.26%, respectively, and 1.81% and 0.76%, respectively, for *P. decipiens*. Meanwhile, Investigator B identified CV values of 0.68% (1 x LOD) and 0.28% (5 x LOD) for *A. simplex* and 1.61% (1 x LOD) and 2.24% (5 x LOD) for *P. decipiens*, with each data point representing the average of the triplicate (Table 5).

In both experiments, no amplification reaction was observed in the negative control (NC), indicating that the PCR was working properly. The CV values of 1xLOD and 5xLOD were less than 4%; therefore, they were judged repeatable and reproducible.

Table 5 Inner-cross checking of anisakids through researcher A and B using real-time PCR

		Mean		SD		CV	
		**A**	**B**	**A**	**B**	**A**	**B**
A. simplex	1 x LOD	32.62	32.44	0.16	0.22	0.50	0.68
	5 x LOD	30.24	30.09	0.08	0.08	0.26	0.28
P. decipiens	1 x LOD	38.42	37.50	0.69	0.60	1.81	1.61
	5 x LOD	36.43	36.55	0.22	0.82	0.76	2.24

CV; SD/Mean

Conclusion

Anisakids are typically identified using traditional morphological identification; however, achieving accurate genus identification is often challenging using the naked eye [9]. Therefore, a genetic approach with high accuracy is required to address this challenge effectively.

Among other molecular biology techniques, real-time PCR has been applied as a rapid detection method to a variety of microorganisms, including bacteria, viruses, and parasites due to its speed, accuracy, and sensitivity [22–25].

Recently, qPCR methods targeting various regions have been developed for the detection of Anisakis [2,16,18–20,26,27]. In this study, we designed our own primer/probe set targeting the cytochrome c oxidase subunit II (COX2) region to develop a more stable detection method that targets COX2 in several mitochondrial genomes.

In summary, this study developed an effective real-time PCR detection method for *A. simplex* and *P. decipiens* that has the potential to be standardized. The optimized detection methods were confirmed to exhibit high sensitivity and specificity in the laboratory. Based on this, it is expected that accurate, rapid, and cost-effective identification of anisakids found in foods, such as fish and seafood, will be possible. In addition, a method for detecting stable genes other than mtDNA sequences needs to be developed.

Acknowledgements

This study was supported by a grant [No. 23191MFDS013] from the Ministry of Food and Drug Safety (MFDS). The findings and conclusions of this study are our own, and do not necessarily represent the views of the Ministry of Food and Drug Safety. We would also like

to thank the Cetacean Research Institute of the National Institute of Fisheries Science for providing *A. simplex* and *P. decipiens* specimens.

Conflict of interest

The authors declare that there is no conflict of interest.

Funding

This study was supported by the Ministry of Food and Drug Safety [23191MFDS013] as part of the Ministry's R&D Project.

References

1. Aibinu IE, Smooker PM, Lopata AL. Anisakis Nematodes in Fish and shellfish- from infection to allergies. Int J Parasitol Parasites Wildl. Internet. 2019;9: 384–393. doi: 10.1016/j.ijppaw.2019.04.007.

2. Molina-Fernández D, Adroher FJ, Benítez R. A scanning electron microscopy study of Anisakis physeteris molecularly identified: from third stage larvae from fish to fourth stage larvae obtained in vitro. Parasitol Res. 2018;117: 2095-2103. doi: 10.1007/s00436-018-5896-5.

3. Baird FJ, Gasser RB, Jabbar A, Lopata AL. Foodborne anisakiasis and allergy. Mol Cell Probes. 2014;28: 167-174. doi: 10.1016/j.mcp.2014.02.003.

4. Nieuwenhuizen NE, Lopata AL. Anisakis--a food-borne parasite that triggers allergic host defences. Int J Parasitol. 2013;43: 1047-1057. doi: 10.1016/j.ijpara.2013.08.001.

5. Quiazon KM, Yoshinaga T, Ogawa K. Distribution of Anisakis species larvae from fishes of the Japanese waters. Parasitol Int. 2011;60: 223-226. doi: 10.1016/j.parint.2011.03.002.

6. Suzuki J, Murata R, Kodo Y. Current status of anisakiasis and Anisakis larvae in Tokyo, Japan. Food Saf (Tokyo). 2021;9: 89-100. doi: 10.14252/foodsafetyfscj.D-21-00004.

7. Sugiyama H, Shiroyama M, Yamamoto I, Ishikawa T, Morishima Y. Anisakiasis annual incidence and causative species, Japan, 2018-2019. Emerg Infect Dis. 2022;28: 2105-2108. doi: 10.3201/eid2810.220627.

8. Choi SH, Kim J, Jo JO, Cho MK, Yu HS, Cha HJ, et al. Anisakis simplex larvae: infection status in marine fish and cephalopods purchased from the Cooperative Fish Market in Busan, Korea. Korean J Parasitol. 2011;49: 39-44. doi: 10.3347/kjp.2011.49.1.39.

9. Sohn WM, Kang JM, Na BK. Molecular analysis of Anisakis type I larvae in marine fish from three different sea areas in Korea. Korean J Parasitol. 2014;52: 383-389. doi: 10.3347/kjp.2014.52.4.383.

10. Sohn WM, Na BK, Kim TH, Park TJ. Anisakiasis: report of 15 gastric cases caused by Anisakis Type I larvae and a brief review of Korean anisakiasis cases. Korean J Parasitol. 2015;53: 465-470. doi: 10.3347/kjp.2015.53.4.465.

11. Adroher-Auroux FJ, Benítez-Rodríguez R. Anisakiasis and Anisakis: an underdiagnosed emerging disease and its main etiological agents. Res Vet Sci. 2020;132: 535-545. doi: 10.1016/j.rvsc.2020.08.003.

12. Nieuwenhuizen NE. Anisakis - immunology of a foodborne parasitosis. Parasite Immunol. 2016;38: 548-557. doi: 10.1111/pim.12349.

13. Armentia A, Santos J, Serrano Z, Martín B, Martín S, Barrio J, et al. Molecular diagnosis of allergy to Anisakis simplex and Gymnorhynchus gigas fish parasites. Allergol Immunopathol (Madr). 2017;45: 463-472. doi: 10.1016/j.aller.2016.12.008.

14. Buchmann K, Mehrdana F. Effects of anisakid nematodes Anisakis simplex (s.l.), Pseudoterranova decipiens (s.l.) and contracaecum osculatum (s.l.) on fish and consumer health. Food Waterborne Parasitol. Internet. 2016;4: 13–22. doi: 10.1016/j.fawpar.2016.07.003.

15. Takano T, Iwaki T, Waki T, Murata R, Suzuki J, Kodo Y, et al. Species composition and infection levels of Anisakis (Nematoda: Anisakidae) in the skipjack tuna Katsuwonus pelamis (Linnaeus) in the northwest Pacific. Parasitol Res. 2021;120: 1605-1615. doi: 10.1007/s00436-021-07144-5.

16. Mossali C, Palermo S, Capra E, Piccolo G, Botti S, Bandi C, et al. Sensitive detection and quantification of anisakid parasite residues in food products. Foodborne Pathog Dis. 2010;7: 391-397. doi: 10.1089/fpd.2009.0428.

17. Paoletti M, Mattiucci S, Colantoni A, Levsen A, Gay M, Nascetti G. Species-specific Real Time-PCR primers/probe systems to identify fish parasites of the genera Anisakis, Pseudoterranova and Hysterothylacium (Nematoda: Ascaridoidea). Fish Res. Internet. 2018;202: 38–48. doi: 10.1016/j.fishres.2017.07.015.

18. Lopez I, Pardo MA. Evaluation of a real-time polymerase chain reaction (PCR) assay for detection of Anisakis simplex parasite as a food-borne allergen source in seafood products. J Agric Food Chem. 2010;58: 1469-1477. doi: 10.1021/jf903492f.

19. Herrero B, Vieites JM, Espiñeira M. Detection of anisakids in fish and seafood products by real-time PCR. Food Control. 2011;22: 933-939. doi: 10.1016/j.foodcont.2010.11.028.

20. Fang W, Liu F, Zhang S, Lin J, Xu S, Luo D. Anisakis pegreffii: a quantitative fluorescence PCR assay for detection in situ. Exp Parasitol. 2011;127: 587-592. doi: 10.1016/j.exppara.2010.11.008.

21. Food Microbiology Division of National Institute of Food and Drug Safety Evaluation, Guidance Documents of Food Poisoning, National Institute of Food and Drug Safety Evaluation. Cheongju; 2023.

22. Hierro N, Esteve-Zarzoso B, González A, Mas A, Guillamón JM. Real-time quantitative PCR (QPCR) and reverse transcription-QPCR for detection and enumeration of total yeasts in wine. Appl Environ Microbiol. 2006;72: 7148-7155. doi: 10.1128/AEM.00388-06.

23. Haramoto E, Kitajima M, Hata A, Torrey JR, Masago Y, Sano D, et al. A review on recent progress in the detection methods and prevalence of human enteric viruses in water. Water Res. 2018;135: 168-186. doi: 10.1016/j.watres.2018.02.004.

24. Sazed SA, Kibria MG, Alam MS. An optimized real-time qPCR method for the effective detection of human malaria infections. Diagnostics (Basel). 2021;11: 736. doi: 10.3390/diagnostics11050736.

25. Fan X, Li X, Zhang T, Xu J, Shi Z, Wu Z, et al. A novel qPCR method for the detection of lactic acid bacteria in fermented milk. Foods. 2021;10: 3066. doi: 10.3390/foods10123066.

26. Łopieńska-Biernat E, Stryiński R, Paukszto Ł, Jastrzębski JP, Makowczenko K. The

selection of reliable reference genes for RT-qPCR analysis of Anisakis simplex sensu stricto gene expression from different developmental stages. Acta Parasitol. 2020;65: 837-842. doi: 10.2478/s11686-020-00220-3.

27. Godínez-González C, Roca-Geronès X, Cancino-Faure B, Montoliu I, Fisa R. Quantitative SYBR Green qPCR technique for the detection of the nematode parasite Anisakis in commercial fish-derived food. Int J Food Microbiol. 2017;261: 89-94. doi: 10.1016/j.ijfoodmicro.2017.05.012.

28. Chalmers RM, Robertson LJ, Dorny P, Jordan S, Kärssin A, Katzer F, et al. Parasite detection in food: current status and future needs for validation. Trends Food Sci Technol. Internet. 2020;99: 337–350. doi: 10.1016/j.tifs.2020.03.011.

Figure 1

Click here to access/download;Figure;Fig. 1.jpg

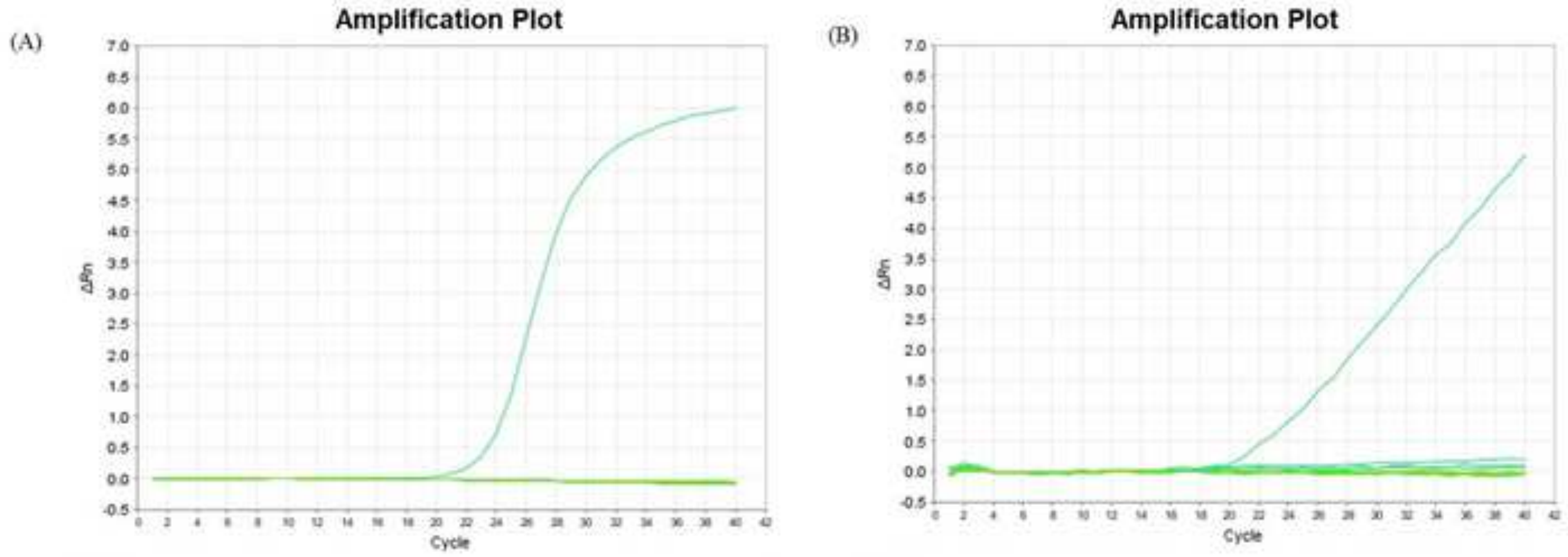

Fig. 1 Specificity for optimized and Real-time PCR methods to detect anisakids

The amplification curves of ascaridoid species was verified using the other 9 parasites. (A) *A. simplex* (B) *P. decipiens*

Figure 2

Click here to access/download;Figure;Fig. 2.jpg

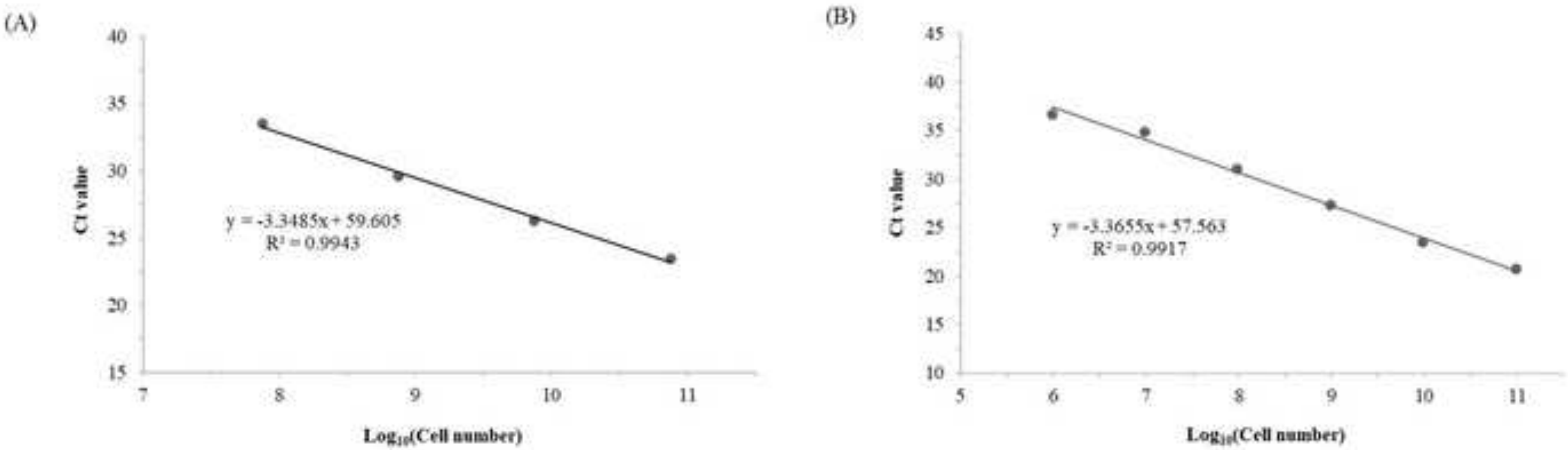

Fig. 2 Standard curve of the Ct value and logarithm of *A. simplex* and *P. decipiens* cell numbers

Linearity test of 10-fold diluted series of (A) *A. simplex* and (B) *P. decipiens* DNA samples
