# Supplementary material for: Comparative study of Taqman-based qPCR assay for the detection of Anisakis simplex and Pseudoterranova decipiens
Source: PLoS One. 2025 Apr 25;20(4):e0320724. doi: 10.1371/journal.pone.0320724 (PMC12027023; doi:10.1371/journal.pone.0320724)
Supplement: Attachment — Submitted filename: Plos one 2024.docx [file pone.0320724.s003.docx]

The manuscript (PONE-D-24-33370) entitled "Comparative study of Taqman-based real-time PCR assay for the detection of *Anisakis simplex* and *Pseudoterranova decipiens* is a interesting article that addresses the important issue of obtaining method detection was that are critical for the evaluation to control present of Anisakid in food.

Parasites family Anisakidae are fish-borne parasites that are responsible for a large number of human infections and allergic reactions around the world. World health organizations and food safety authorities aim to control and prevent this emerging health problem. Specially *Anisakis simplex* and *Pseudoterranova decipiens* are main dangerous for human health. A rapid and sensitive detection strategy (DNA, protein) can provide authorities with an effective and fast method to guarantee consumer safety, therefore research and methods are particularly important.

**Comments:**

**Line 36** Anisakiasis, is a name of diseases cause only nematode parasite belong to genus Anisakis

**Line 37:** “third-instar” is description of insect larval stage!!!!!! https://doi.org/10.1016/B978-0-12-820359-0.00002-5.

**Line 31 and 51, 54** Anisakis, precise meaning - genus? mistake – Anisakids? And many times, the same in whole manuscript!!!!

**Line 61** remove” infected” repetition

**Line 94,98** only larvae of Anisakis not Pseudoterranova???

**Line 123,** how reference methods? sspecific papers?

**Line 124,** molecular material, template is DNA. Where does cDNA synthesis come from? The material, template was RNA?

What are the stages of PCR? Basic knowledge, denaturation, annealing, extension……

**Line 129, 130 etc.** What 9 parasites? What 2 larvae?

**Line 146,** 3 d? 3 days? 72 hours?

**Line 164,** What ascaridoid species?

**Line 220,** cell numbers? cell of plate?

In the "**Results and discussion**" in my opinion it is necessary to compare and clearly present the protocol for using the method sensitivity demonstrated for the authors!

Little Explanation:

Anisakis – genus

Anisakiosis/Anisakiasis - disease cause for Anisakis genus

Anisakidae family (Anisakis, Pseudoteranova, Contraceucum)

Anisakidosis disease cause for family Anisakidae (Anisakis, Pseudoterranova, Contraceucum)

Anisakids, English colloquial, customary, non-taxonomic!!!using in papers

fish-borne parasites belong to family Anisakidae

Detection DNA from larvae, have there been any attempts to detect DNA of parasite from tissue of fish?

As the authors write, there are many papers (2, 16, 18) on the detection of Nematodes belong to Anisakidae, there is a lack of discussion with other authors, and above all, there is no demonstration of higher sensitivity of the method validation by the authors, a protocol for further research, and use in detection in food.

How aim of this work?

The paper has many mistakes substantive, knowledge of taxonomy, nomenclature,

deficiencies in the methodology and ambiguities in the description of the method.

The work is scientifically sound and might to consideration for publication after thorough improvement. The experimental design is correct but not Research Paper maybe as a “Submissions describing **methods** and **validation- protocol?”**
