# Supplementary material for: Comparative study of Taqman-based qPCR assay for the detection of Anisakis simplex and Pseudoterranova decipiens
Source: PLoS One. 2025 Apr 25;20(4):e0320724. doi: 10.1371/journal.pone.0320724 (PMC12027023; doi:10.1371/journal.pone.0320724)
Supplement: Attachment — Submitted filename: (20241025) Response to Reviewers_PONE-D-24-33370-final.docx [file pone.0320724.s005.docx]

**PONE-D-24-33370**

**Comparative study of Taqman-based real-time PCR assay for the detection of Anisakis simplex and Pseudoterranova decipiens**

**A point-to-point response to reviewers’ comments**

We are very grateful to the helpful and insightful comments from Journal Requirements, Reviewer #1 #2, #3, and #4 have carefully revised our manuscript accordingly.

- **First of all, thank you for providing many professional opinions despite your busy schedule. Hello, I am Seo Doo Won, the corresponding author of this paper, from the Microbiology Division of the Korea Food and Drug Administration, conducting research related to food safety management in Korea. We have conducted a study to analyze foodborne pathogens, particularly parasites, that may arise due to climate change and dietary habits involving the consumption of raw foods. In this study, we aimed to identify optimal genetic testing methods for detecting parasites that may cause acute or chronic infections in foods of concern in the future, based on a review and comparison of domestic and international literature on real-time genetic analysis methods. We believe that the real-time PCR method can provide faster results than conventional PCR methods, which is why we chose this approach for our research. Additionally, the PCR composition was based on methods previously used by the Korea Food and Drug Administration for genetic testing of parasites during foodborne illness investigations.**

**Regarding the target of this paper, Anisakis simplex, there have been reports of it in food in Korea in 2017, 2019, and 2023. Therefore, as the reviewers mentioned, it would be very challenging to analyze actual samples from food. Consequently, we determined that it would be appropriate to use samples obtained from another research institution in Korea, the Whale Research Institute, which included five samples of Anisakis simplex and two samples of Pseudoterranova decipiens.**

Below is our point-by-point response to each comment.

**[Journal Requirement ]**

Commnet :

**1. Please ensure that your manuscript meets PLOS ONE's style requirements, including those for file naming. The PLOS ONE style templates can be found at**

**https://journals.plos.org/plosone/s/file?id=wjVg/PLOSOne_formatting_sample_main_body.pdf and**

**https://journals.plos.org/plosone/s/file?id=ba62/PLOSOne_formatting_sample_title_authors_affiliations.pdf**

• **Response:**

I have revised the text according to the two guidelines (Title, Author, Affiliations Formatting Guidelines, Manuscript Body Formatting Guidelines).

Commnet :

**2.Thank you for stating the following in the Acknowledgments Section of your manuscript:**

**"This study was supported by a grant [No. 23191MFDS013] from the Ministry of Food and Drug Safety (MFDS). The findings and conclusions of this study are our own, and do not necessarily represent the views of the Ministry of Food and Drug Safety. We would also like to thank the Cetacean Research Institute of the National Institute of Fisheries Science for providing A. simplex and P. decipiens specimens."**

**We note that you have provided additional information within the Acknowledgements Section that is not currently declared in your Funding Statement. Please note that funding information should not appear in the Acknowledgments section or other areas of your manuscript. We will only publish funding information present in the Funding Statement section of the online submission form.**

**Please remove any funding-related text from the manuscript and let us know how you would like to update your Funding Statement. Currently, your Funding Statement reads as follows:**

**"This study was supported by the Ministry of Food and Drug Safety as part of the Ministry's R&D project"**

**Please include your amended statements within your cover letter; we will change the online submission form on your behalf.**

• **Response:**

**We've made the following changes based on your suggestions.**

**This study was supported by the Ministry of Food and Drug Safety as part of the Ministry's R&D project**

Commnet :

**3. We note that the grant information you provided in the ‘Funding Information’ and ‘Financial Disclosure’ sections do not match.**

**When you resubmit, please ensure that you provide the correct grant numbers for the awards you received for your study in the ‘Funding Information’ section.**

• **Response:**

**I will revise it to align with the funding information/financial disclosure.**

Commnet :

**4. Thank you for stating the following financial disclosure:**

**"This study was supported by the Ministry of Food and Drug Safety as part of the Ministry's R&D project."**

**Please state what role the funders took in the study. If the funders had no role, please state: "The funders had no role in study design, data collection and analysis, decision to publish, or preparation of the manuscript."**

**If this statement is not correct you must amend it as needed.**

**Please include this amended Role of Funder statement in your cover letter; we will change the online submission form on your behalf.**

• **Response:**

**The institution that authored the paper (the Korea Institute of Food and Drug Safety Evaluation) is an affiliated organization of the Ministry of Food and Drug Safety. Accordingly, while it is correct that the budget was provided by the Ministry of Food and Drug Safety, as you inquired, it had no role in the design of the research, data collection, analysis, decision to publish the paper, or preparation of the manuscript.**

Commnet :

**5. When completing the data availability statement of the submission form, you indicated that you will make your data available on acceptance. We strongly recommend all authors decide on a data sharing plan before acceptance, as the process can be lengthy and hold up publication timelines. Please note that, though access restrictions are acceptable now, your entire data will need to be made freely accessible if your manuscript is accepted for publication. This policy applies to all data except where public deposition would breach compliance with the protocol approved by your research ethics board. If you are unable to adhere to our open data policy, please kindly revise your statement to explain your reasoning and we will seek the editor's input on an exemption. Please be assured that, once you have provided your new statement, the assessment of your exemption will not hold up the peer review process.**

• **Response:**

**The experimental result data can be accessed through the research management system of the Ministry of Food and Drug Safety.**

**[Reviewer #1]**

**※ Comments:**

**The MS presented to me for review is well attempted effort to develop a new Taqman-based real-time PCR assay for the detection of Anisakis simplex and Pseudoterranova decipiens. However, introduction should have included extent of the problem due to raw fish eating and population susceptible. Apart from this, the development is based on DNA extraction from Anisakis larvae provided apart from other larvae and parasitic species obtained. I would have wish to see, if they could have further tested the diagnostic probe in suspected food samples likely to be consumed by human. Rest others seems Ok to me and few marked in MS.**

• **Response:**

**First of all, thank you for your valuable professional feedback despite your busy schedule. Hello, I am Seo Doo Won, the corresponding author of this paper, and I am conducting research related to food safety management in Korea. We have been studying foodborne pathogens, particularly parasites, in relation to climate change and the consumption of raw foods. In this research, we aimed to find the optimal method for detecting acute or chronic infections caused by parasites in foods that are not frequently reported in Korea but are of concern for the future. We investigated and compared gene testing methods reported both domestically and internationally (real-time gene analysis methods).**

**We believe that real-time PCR methods would provide faster results than conventional PCR methods, which is why we chose this approach for our study. Additionally, the PCR composition was designed considering the testing conditions (PCR composition) used by the National Institute of Food and Drug Safety Evaluation for investigating foodborne pathogens.**

**Regarding the target of this paper, Anisakis simplex, there have been reported cases in food in Korea in 2017, 2019, and 2023. Therefore, as the reviewers mentioned, analyzing samples from actual food would be very challenging. Consequently, we deemed it appropriate to use samples isolated from Anisakis simplex and Pseudoterranova decipiens obtained from the Korea Institute of Whale Research, another research institution in Korea.**

**※ Comments:**

It should include brief about habit of raw fish eating in the area and global context and suspected population to be affected?

• **Response:**

Lines :

**-> The consumption of anisakid larvae generally results in a self-limiting infection that sensitizes individuals to Anisakis allergens. Japan has the highest rate of Anisakis infections among humans, with infected sushi and sashimi—traditional dishes featuring raw fish—being major sources of these infections. Annually, there are between 2,000 and 3,000 reported cases of anisakiasis. The globalization of local cuisines has also led to an increase in the number of people consuming raw fish[29].**

**※ Comments:**

**line 62 :** Marine mammal or only whale fish?

• **Response:**

Lines : **marine mammal**

**※ Comments:**

**line 79 :** rewrite for clarity?

Recently, *A. simplex* was reported to increase the prevalence of hypersensitivity in freezing fish [13].

• **Response:**

Lines : **Recently, it was reported that A. simplex is associated with a higher prevalence of hypersensitivity in frozen fish[13]**

**※ Comments:**

**line 88 :**After development of probe for detection based on larvae samples obtained, I would have wished to see it being conducted in actual cause of trouble in the human population, i.e suspected uncooked marine sea foods?

• **Response:**

Lines :

**As mentioned earlier, the occurrence of whale worms in food is extremely low, so no analysis from actual food sources was conducted.**

**※ Comments:**

**line 90-92** temperature of storage?

Anisakis larvae isolated from marine mammals were provided by the Cetacean Research Institute, National Institute of Fisheries Science, and stored in 100% ethanol until DNA extraction.

• **Response:**

Lines :

**Anisakis and Pseudoterranova larvae isolated from marine mammals were provided by the Cetacean Research Institute, National Institute of Fisheries Science, and stored in 100% ethanol until DNA extraction at room temperature**

**※ Comments:**

line 242 : better as molecular

Therefore, a genetic approach with high accuracy is required to address this challenge effectively.

• **Response:**

Lines : **Therefore, a molecular approach with high accuracy is required to address this challenge effectively.**

**※ Comments:**

**line 247-249 :** Apart from new probe set reported for detection, how it is better or limitation from other probes reported earlier? Where earlier qPCR methods suffering from stability in detection or non-specific or less sensitive.

• **Response:**

Lines :

**As mentioned earlier, it has not been confirmed whether there were issues with the existing testing methods. The aim was to compare the sensitivity of the existing methods through a comparative analysis to identify a better testing method for the parasite in question.**

**※ Comments:**

**ine 252-255 :** It was not attempted in suspected sea food consumed raw and also its possibility in detection in semi-cooked sea fish could have been attempted?

Based on this, it is expected that accurate, rapid, and cost-effective identification of anisakids found in foods, such as fish and seafood, will be possible. In addition, a method for detecting stable genes other than mtDNA sequences needs to be developed.

• **Response:**

Lines :

**As mentioned earlier, it has not been confirmed whether there were issues with the existing testing methods. The aim was to compare the sensitivity of the existing methods through a comparative analysis to identify a better testing method for the parasite in question.**

**[Reviewer #2]**

**※ Comments:**

**The manuscript (PONE-D-24-33370) entitled "Comparative study of Taqman-based real-time PCR assay for the detection of Anisakis simplex and Pseudoterranova decipiens is a interesting article that addresses the important issue of obtaining method detection was that are critical for the evaluation to control present of Anisakid in food.**

**Parasites family Anisakidae are fish-borne parasites that are responsible for a large number of human infections and allergic reactions around the world. World health organizations and food safety authorities aim to control and prevent this emerging health problem. Specially Anisakis simplex and Pseudoterranova decipiens are main dangerous for human health. A rapid and sensitive detection strategy (DNA, protein) can provide authorities with an effective and fast method to guarantee consumer safety, therefore research and methods are particularly important.**

• **Response:**

**First of all, thank you for your valuable professional feedback despite your busy schedule. Hello, I am Seo Doo Won, the corresponding author of this paper, and I am conducting research related to food safety management in Korea. We have been studying foodborne pathogens, particularly parasites, in relation to climate change and the consumption of raw foods. In this research, we aimed to find the optimal method for detecting acute or chronic infections caused by parasites in foods that are not frequently reported in Korea but are of concern for the future. We investigated and compared gene testing methods reported both domestically and internationally (real-time gene analysis methods).**

**We believe that real-time PCR methods would provide faster results than conventional PCR methods, which is why we chose this approach for our study. Additionally, the PCR composition was designed considering the testing conditions (PCR composition) used by the National Institute of Food and Drug Safety Evaluation for investigating foodborne pathogens.**

**Regarding the target of this paper, Anisakis simplex, there have been reported cases in food in Korea in 2017, 2019, and 2023. Therefore, as the reviewers mentioned, analyzing samples from actual food would be very challenging. Consequently, we deemed it appropriate to use samples isolated from Anisakis simplex and Pseudoterranova decipiens obtained from the Korea Institute of Whale Research, another research institution in Korea.**

**※ Comments:**

**Line 36 Anisakiasis, is a name of diseases cause only nematode parasite belong to genus Anisakis**

• **Response:**

Lines : **Anisakidosis**

**※ Comments:**

**Line 37: “third-instar” is description of insect larval stage!!!!!!** [**https://doi.org/10.1016/B978-0-12-820359-0.00002-5.**](https://doi.org/10.1016/B978-0-12-820359-0.00002-5.)

• **Response:**

Lines : **third stage larvae**

**※ Comments:**

**Line 31 and 51, 54 Anisakis, precise meaning - genus? mistake – Anisakids? And many times, the same in whole manuscript!!!!**

• **Response:**

Lines : **Anisakidosis**

**※ Comments:**

**Line 61 remove” infected” repetition**

• **Response:**

Lines : **Anisakiasis, a zoonotic disease that occurs worldwide, is caused by the larvae of nematodes that parasitize the stomachs of marine mammals, such as whales, dolphins, and seals. Anisakiasis is a parasitic infectious disease transmitted to humans by consuming infected raw fish** ~~infected~~

**※ Comments:**

**Line 94,98 only larvae of Anisakis not Pseudoterranova???**

• **Response:**

Lines : **DNA was extracted by cutting a 1-cm section of the body of a single** Anisakis simplex & pseudoterranova decipiens larva

**※ Comments:**

**Line 123, how reference methods? sspecific papers?**

• **Response:**

Lines : **The PCR conditions were adapted from the respective** reference methods[21]

**※ Comments:**

**Line 124, molecular material, template is DNA. Where does cDNA synthesis come from? The material, template was RNA?**

**What are the stages of PCR? Basic knowledge, denaturation, annealing, extension……**

• **Response:**

Lines : **line 117 : The real-time PCR mixtures for each method were Taqman Universal Master mix II** with UNG

**line 124 : UNG activation ~~cDNA synthesis~~** **at 50 °C for 2 min**, Polymerase activation ~~pre-heating~~ at **95 °C for 10 min, denaturation at 95 °C for 15 s for 40 cycles, and annealing and extension at 57– 61 °C for 1 min**

**※ Comments:**

**Line 129, 130 etc. What 9 parasites? What 2 larvae?**

• **Response:**

Lines : **eight other species parasites(Clonorchis sinensis, Toxoplasma gondii, Metagonimus yokogawai, Gymnophalodides seoi, Taenia solium, Anisakis physeteris, Contracaecum osculatum, and Pseudoterranova azarasi) and two larvae(A.simplex and P.decipiens)**

**※ Comments:**

**Line 146, 3 d? 3 days? 72 hours?**

• **Response:**

Lines : **Inter-examiner validation of the method was performed by two investigators in our laboratory once daily for a total of 3 days**

**※ Comments:**

**Line 164, What ascaridoid species?**

• **Response:**

Lines :**anisakidae family**

**※ Comments:**

**Line 220, cell numbers? cell of plate?**

**In the "Results and discussion" in my opinion it is necessary to compare and clearly present the protocol for using the method sensitivity demonstrated for the authors!**

• **Response:**

Lines **: Fig.2 Standard curve of the Ct value and logarithm of A.simplex and P.decipiens DNA numbers**

**Little Explanation:**

**Anisakis – genus**

**Anisakiosis/Anisakiasis - disease cause for Anisakis genus**

**Anisakidae family (Anisakis, Pseudoteranova, Contraceucum)**

**Anisakidosis disease cause for family Anisakidae (Anisakis, Pseudoterranova, Contraceucum)**

**Anisakids, English colloquial, customary, non-taxonomic!!!using in papers**

**fish-borne parasites belong to family Anisakidae**

**Detection DNA from larvae, have there been any attempts to detect DNA of parasite from tissue of fish?**

**As the authors write, there are many papers (2, 16, 18) on the detection of Nematodes belong to Anisakidae, there is a lack of discussion with other authors, and above all, there is no demonstration of higher sensitivity of the method validation by the authors, a protocol for further research, and use in detection in food.**

**How aim of this work?**

**The paper has many mistakes substantive, knowledge of taxonomy, nomenclature,**

**deficiencies in the methodology and ambiguities in the description of the method.**

**※ Comments:**

• **Response:**

Lines :

**-> revised conclusion**

**We planned a study to analyze potential foodborne pathogens related to food consumption habits and climate change using real-time PCR methods. Accordingly, we aimed to investigate and compare genetic testing methods for parasites that may cause acute or chronic infections in food items, which are not frequently reported in South Korea but are a concern for the future. This was done by reviewing domestic and international research papers to identify the optimal genetic testing method.**

**In particular, we believed that real-time PCR would yield faster results compared to conventional PCR methods, so we conducted our research using this approach. Additionally, we considered the PCR composition methods utilized by the Korea Food and Drug Administration for genetic testing of parasites during foodborne illness investigations.**

**As a result of comparing genetic testing methods for detecting two types of parasites, we were able to identify a method with high sensitivity. This method is expected to assist in the future identification of these two parasites using genetic amplification techniques.**

**[Reviewer #3]**

**First of all, thank you for your valuable professional feedback despite your busy schedule. Hello, I am Seo Doo Won, the corresponding author of this paper, and I am conducting research related to food safety management in Korea. We have been studying foodborne pathogens, particularly parasites, in relation to climate change and the consumption of raw foods. In this research, we aimed to find the optimal method for detecting acute or chronic infections caused by parasites in foods that are not frequently reported in Korea but are of concern for the future. We investigated and compared gene testing methods reported both domestically and internationally (real-time gene analysis methods).**

**We believe that real-time PCR methods would provide faster results than conventional PCR methods, which is why we chose this approach for our study. Additionally, the PCR composition was designed considering the testing conditions (PCR composition) used by the National Institute of Food and Drug Safety Evaluation for investigating foodborne pathogens.**

**Regarding the target of this paper, Anisakis simplex, there have been reported cases in food in Korea in 2017, 2019, and 2023. Therefore, as the reviewers mentioned, analyzing samples from actual food would be very challenging. Consequently, we deemed it appropriate to use samples isolated from Anisakis simplex and Pseudoterranova decipiens obtained from the Korea Institute of Whale Research, another research institution in Korea.**

**※ Comments:**

**Comparative study of Taqman-based real-time PCR assay for the detection of Anisakis simplex and Pseudoterranova decipiens**

**Authors: Mi-Gyeong Kim, Min Ji Hong, Doo Won Seo, Hyun Mi Jung, Hyun-Ja Han, Seung Hwan Kim, Insun Joo**

**General comments:**

**This manuscript presents a comparative study of a TaqMan-based real-time PCR assay for the detection of two heteroxenous parasites, Anisakis simplex and Pseudoterranova decipiens. These parasites are among the most significant biological hazards in seafood products, causing Anisakiasis in humans. Accurate detection is therefore crucial for ensuring food safety and protecting public health.**

**However, the manuscript requires significant improvements before it can be considered for publication. Specifically, the writing needs enhancement, particularly in the Results and Discussion sections. It is important to clearly differentiate between the findings and provide a detailed discussion of each result. Expanding the Results and Discussion sections will make the manuscript more robust and suitable for publication. Additionally, the Materials and Methods section should be elaborated to ensure that readers can easily understand the experimental procedures.**

**For the qPCR section, it is recommended to adhere to the Minimum Information for Publication of Quantitative Real-Time PCR Experiments (MIQE) guidelines (Bustin et al., 2009) and use the appropriate terminology to avoid any confusion. Including the MIQE checklist in the supplementary data would also be beneficial.**

**References**

**Bustin, S.A., Benes, V., Garson, J.A., Hellemans, J., Huggett, J., Kubista, M., Mueller, R., Nolan, T., Pfaffl, M.W., Shipley, G.L., 2009. The MIQE guidelines: minimum information for publication of quantitative real-time PCR experiments. Clin. Chem. 55, 611–622**

**Other comment and suggestions: -**

**Introduction: It is suggested to include previous studies that employed different approaches for detecting Anisakis simplex and Pseudoterranova decipiens, or other members of the Anisakidae family, such as multiplex PCR or alternative primers. Discussing the limitations of these previous studies and the rationale behind designing the current study would enhance the reader's understanding of the study's background and objectives.**

**※ Comments:**

**Line 99: Clarification is needed regarding whether the research data mentioned here refers to data from previous studies on domestic and foreign parasite genes. If it does, please provide the appropriate citations. Additionally, it would be more informative to specify the types of data reviewed (e.g., sequences, detection protocols) and the criteria used to select relevant methods.**

• **Response:**

Lines :

**-> Before constructing the primer and probe sets for Anisakis detection, we collected and analyzed research data on domestic and foreign parasite gene detection methods and cataloged the primers/probes for the parasite[15, 16, 17, 18 ,19 ,21]**

**※ Comments:**

**Line 103: The term "not cross-detected" could be made more explicit by specifying the criteria used, such as the specific thresholds or parameters applied in the BLAST search.**

• **Response:**

Lines :

**※ Comments:**

**Line 105: Please provide citations for the literature referenced in this section.**

• **Response:**

Lines : **In addition to those obtained from the literature[21, 15, 16, 17, 18, 19], we designed primers/probes for verification.**

**※ Comments:**

**Line 106-107: Please provide the accession numbers for all taxon sampling sequences. These can be included in the supplementary data.**

• **Response:**

Lines :

**The sequences of A. simplex[KC810002.1] and P. decipiens[OK338708] mtDNA cox2 obtained from the National Center for Biotechnology Information were aligned using the Mega 11 program**.

**※ Comments:**

**Line 109: Please provide a citation or link for "Genscript." If available, include the version used as well.**

**Section Design Primers and Probe: Clarification is needed on whether the designed primers and probes were validated through in silico analysis for primer specificity using the NCBI database.**

• **Response:**

Lines :

**.**

**The most common sequence resulting from the alignment was used as the template sequence (A. simplex (LC543759), P. decipiens (AB576757.1)) and subjected to Genscript(https://www.genscript.com/) (optimal primer size: 20 bp, optimal primer Tm: 60.0 ℃, optimal product size: 200 bp), and IDT(https://sg.idtdna.com/) (primer size: 17–30 bp, primer Tm: 59-65 ℃, primer GC content: 35–65%, product size: 75–150 bp) using the homepage services of Genscript (optimal primer size: 20 bp, optimal primer Tm: 60).**

**※ Comments:**

**Line 123: Clarification is needed regarding what the reference methods refer to. Providing the exact references or more details on how these methods were adapted (e.g., changes in cycling conditions, reagent concentrations) would improve transparency and allow others to replicate or build on the work.**

• **Response:**

Lines :

**-> The PCR conditions were adapted from the respective reference methods[21]**

**※ Comments:**

**Line 126: Clarification is needed on whether the study considered a result valid and if any quality control measures were applied, such as the inclusion of no-template controls or positive controls.**

• **Response:**

Lines : **> Each real-time PCR sample was analyzed in duplicate with no-template control.**

**※ Comments:**

**Line 129: Please specify the nine other parasite species and the two larvae used to confirm non-specific detection. Are these species closely related to Anisakis and Pseudoterranova, or were they selected because they are commonly found in similar environments? Explaining the rationale behind the selection of these particular species would be beneficial.**

• **Response:**

Lines :

**As mentioned earlier, the goal was to detect parasites that pose a risk of foodborne illness through genetic testing. Therefore, in addition to Anisakis simplex and Pseudoterranova decipiens, the analysis included actual parasite species such as Clonorchis sinensis, Toxoplasma gondii, Metagonimus yokogawai, Gymnophalodides seoi, and Taenia solium. Additionally, among the five species of whale parasites analyzed, two species had limited reports of isolation from food, so parasites isolated through distribution agencies were used for the analysis.**

**※ Comments:**

**Line 133: Please clarify whether these plasmids contain full-length gene sequences or specific target regions, and whether any controls were used to ensure the accuracy of these plasmid constructs.**

**Additionally, provide details on how the plasmid DNA from Anisakis physeteris, Contracaecum osculatum, and Pseudoterranova azarasi was synthesized. Was this done using a cloning kit? Specify the type of plasmid DNA used in the study and indicate whether the plasmid was linearized using a restriction enzyme (RE).**

**Section Synthesis of Positive Control: This section would benefit from additional details. Please elaborate on how the positive control was synthesized. Was it generated using a PCR amplicon, synthetic gene fragments, or PCR synthesis from a plasmid? Additionally, specify which region was selected for the synthesis.**

**Section Inter-Examiner Validation: Additional details would enhance the clarity and comprehensiveness of this section. Consider the following suggestions:**

**-Briefly explain the purpose of inter-examiner validation.**

**-Clarify the exact concentrations of the dilutions used, specifying them in terms of DNA copies per microliter or nanograms per microliter for precision.**

**-Indicate the number of replications performed.**

**-Specify the statistical tests used to compare the results between the two investigators, such as interclass correlation coefficients or Bland-Altman analysis, to assess agreement or consistency between examiners.**

**Provide the rationale for conducting the validation once daily for three days. Was any longer-term validation (e.g., over weeks or months) planned or conducted?**

• **Response:**

Lines : **->**

**Plasmid DNA from Anisakis physeteris, Contracaecum osculatum, and Pseudoterranova azarasi were used as templates**

**Anisakis physeteris, cox1, 228 bp**

**Contracaecum osculatum, cox1, 804 bp**

**Pseudoterranova azarasi, cox1, 228 bp**

A. simplex	1XLOD: 1.93 ⅹ10^7^ copy/μL	1.9 ⅹ10^7^ ng/μL

5XLOD: 9.65 ⅹ10^7^ copy/μL	9.5 ⅹ10^7^ ng/μL

P. decipiens	1XLOD: 9.67ⅹ10^5^ copy/μL	1.1 ⅹ10^6^ ng/μL

5XLOD: 4.84ⅹ10^6^ copy/μL	5.4 ⅹ10^6^ ng/μL

**※ Comments:**

**Line 153: Please specify the starting year for the 10-year period mentioned.**

• **Response:**

Lines : **->**

**A literature search ~~of the last 10 years~~ for Taqman-based real-time PCR gene detection in anisakid (Table 1) revealed six studies concerning A. simplex and one for P. decipiens [2, 16-20]**

**※ Comments:**

**Line 154: The citation "(Table 1)" would be more appropriately placed at the end of the statement, following "P. decipiens," as in "P. decipiens (Table 1)..."**

• **Response:**

Lines :

**-> -> P.decipiens.(Table 1)**

**※ Comments:**

**Line 155-157: The study's findings should be clearly stated here. It is suggested to rephrase as: "This study designed several sets of primers and probes based on the sequences of..."**

• **Response:**

Lines :

**>**

**I designed primers/probes for real-time PCR studies on Anisakis simplex and Pseudoterranova decipiens, in addition to the primers/probes created in the paper.**

**※ Comments:**

**Line 157: Clarification is needed regarding the "seven detection methods" mentioned. Are these methods referring to all the primer/probe sets used in the qPCR assay, or are they separate techniques? Please rephrase this sentence to avoid confusion.**

• **Response:**

Lines :

**In total, ~~seven detection~~ thirteen methods were selected for the comparison between A. simplex and P. decipiens (Table 1, 2).**

**※ Comments:**

**Line 158-159: The sentence "The detection regions of the selected primer/probe sets were first checked for overlap with those of other Anisakis species and genera using BLAST..." refers to the in-silico analysis of primer/probe specificity. It would be more useful to present the results of this analysis in the manuscript. Specifically, indicate whether all BLAST hits were on the targeted species, and if there were any hits on non-target species. Provide details on the percentage of hits, as well as the similarity and coverage percentages.**

• **Response:**

Lines :

**->**

**※ Comments:**

**Line 160-161: Although the real-time PCR showed a single independent amplification, this alone does not confirm that the detected amplicon is from the targeted region for the species. To ensure accuracy, it would be better to perform sequencing of the PCR or real-time PCR product to confirm the identity of the amplicon.**

• **Response:**

Lines :

**->** **According to Figure 6, the sequencing results for the two species confirmed that they are Anisakis simplex and Pseudoterranova decipiens.**

**※ Comments:**

**Line 178: The term "selected reference" needs clarification. Is it referring to the primers/probes shown in Table 1? Please clearly specify this to help readers better understand the context.**

• **Response:**

Lines :

**-> The sequencing results confirmed that the two species are Anisakis simplex and Pseudoterranova decipiens**

**※ Comments:**

**Line 178-179: The term "PCR conditions" may cause confusion. It is suggested to either refer to the MIQE guidelines for appropriate terminology or replace it with "qPCR cycling conditions" for clarity.**

• **Response:**

Lines :

**-> . The real-time PCR conditions used the qPCR cycling conditions of the selected reference and the parasite gene detection method in the Guidance Documents of Food Poisoning Test [21] (Table 3)**

**※ Comments:**

**Line 180-181: It would be more effective to include the qPCR amplification curve results in this section to provide clearer evidence of the findings.**

• **Response:**

Lines :

**-> The results showed that three instances of A. simplex and five instances of P. decipiens were detected(Fig 3.)**

**※ Comments:**

**Line 186-187: Please include the amplification curve as part of the results for this finding.**

• **Response:**

Lines :

**-> The method with the most stable Ct value (< 30) was selected based on a comparison between methods using actual extracted DNA [16] (Table 3),(Fig4)**

**※ Comments:**

**Line 187-192: The current statement reads more like a description of materials and methods rather than presenting actual results. To improve clarity, rephrase the section by explaining the outcomes shown in Table 4. Additionally, it is recommended to include the amplification curves as part of the result to better illustrate the findings.**

**Table 3: Please include the primer sequences and their corresponding annealing temperatures in the table.**

• **Response:**

Lines :

**-> The primer:probe concentration combinations (400 nM:200 nM, 400 nM:250 nM, 500 nM:200 nM, and 500 nM:250 nM) were applied as PCR conditions for each set. We further optimized the detection method by analyzing qPCR assays with different primer/probe set concentration combinations. The final concentrations selected were 400 nM/250 nM for A. simplex and 400 nM/200 nM for P. decipiens (Table 4).(fig 4)**

**※ Comments:**

**Line 202: Were the primers/probes tested on wild samples?**

• **Response:**

Lines :

**Additionally, this section should include some discussion rather than just presenting the results, as this is the Results and Discussion section.**

**-> In Table 1-2, the sequences are mentioned, and the annealing Tm has been added in Table 3**

**※ Comments:**

**Line 211: It would be beneficial to include the equation for converting copies/µL to ng or cells in the Materials and Methods section. This addition would provide clarity on the calculation process.**

**Fig 2: The statement in lines 201-215 mentions the detection limit in terms of gene copy number (copies/µL), but Figure 2 shows log10 cell number. Please confirm if the figure label is correct. Additionally, clarify the relationship between gene copy number and cell number. What is the gene copy number per cell?**

• **Response:**

Lines :

**-> line 211**

**-> fig 2 was reviesd**

**※ Comments:**

**Line 225: In addition to Table 5, it is crucial to provide the raw data for the amplification curves in the supplementary materials to demonstrate the reliability of these findings.**

• **Response:**

Lines :

**As a result of detection by real-time PCR, the CV values of 1ⅹLOD and 5ⅹLOD of A. simplex gene amplification products of Investigator A were 0.50% and 0.26%, respectively, and 1.81% and 0.76%, respectively, for P. decipiens. Meanwhile, Investigator B identified CV values of 0.68% (1ⅹLOD) and 0.28% (5ⅹLOD) for A. simplex and 1.61% (1ⅹLOD) and 2.24% (5ⅹLOD) for P. decipiens, with each data point representing the average of the triplicate (Table 5).(fig 5)**

**※ Comments:**

**Line 243-249: The statements in this section are more appropriate for the Introduction. Please consider moving them to the Introduction section.**

• **Response:**

Lines :

**-> I have changed it to the introduction section.**

**[Reviewer #4]**

**First of all, thank you for your valuable professional feedback despite your busy schedule. Hello, I am Seo Doo Won, the corresponding author of this paper, and I am conducting research related to food safety management in Korea. We have been studying foodborne pathogens, particularly parasites, in relation to climate change and the consumption of raw foods. In this research, we aimed to find the optimal method for detecting acute or chronic infections caused by parasites in foods that are not frequently reported in Korea but are of concern for the future. We investigated and compared gene testing methods reported both domestically and internationally (real-time gene analysis methods).**

**We believe that real-time PCR methods would provide faster results than conventional PCR methods, which is why we chose this approach for our study. Additionally, the PCR composition was designed considering the testing conditions (PCR composition) used by the National Institute of Food and Drug Safety Evaluation for investigating foodborne pathogens.**

**Regarding the target of this paper, Anisakis simplex, there have been reported cases in food in Korea in 2017, 2019, and 2023. Therefore, as the reviewers mentioned, analyzing samples from actual food would be very challenging. Consequently, we deemed it appropriate to use samples isolated from Anisakis simplex and Pseudoterranova decipiens obtained from the Korea Institute of Whale Research, another research institution in Korea.**

**Reviewer #4: The authors deal with a parasitic nematode of public health importance, Anisakis simplex and Pseudoterranova decipiens. Unfortunately, the paper submitted for review is written in an unreliable manner and raises my doubts.**

**※ Comments:**

**The attempt made by the authors to produce a Taqman probe for the detection of the above-mentioned nematode species in food was, in my opinion, not carried out in accordance with the current standards of molecular biology. For example, qPCR is a quantitative method, and the authors used different amounts of DNA matrix of the tested species for the analyzes, which is a mistake (line 119).**

**In addition, the preparation of the primer sequence and probes is not clearly discussed. There is a lack of alignments in the form of a figure with a discussion or at least in the supplement.**

• **Response:**

Lines :

**-> To compare the genetic testing methods of isolated Anisakis simplex and Pseudoterranova decipiens, DNA preparation was conducted, and the amount of DNA obtained from this process is indicated in line 119. Additionally, regarding the preparation of primers and probes, we selected the primers/probes based on a literature review from domestic and international sources, as stated in lines 100-103, and compiled a list of the selected primers/probes in Tables 1 and 2.**

**At the same time, according to the authors, the analysis of specificity and detection limit was based on nine other parasitic species. I have mentioned 8 other species in the text. Why were these species selected? There are spelling errors in the species names (correct spelling Gymnophalloides seoi).**

**->**

**As mentioned earlier, the goal was to detect parasites that pose a risk of foodborne illness through genetic testing. Therefore, in addition to Anisakis simplex and Pseudoterranova decipiens, the analysis included actual parasite species such as Clonorchis sinensis, Toxoplasma gondii, Metagonimus yokogawai, Gymnophalodides seoi, and Taenia solium. Additionally, among the five species of whale parasites analyzed, two species had limited reports of isolation from food, so parasites isolated through distribution agencies were used for the analysis.**

**Why was plasmid DNA from Anisakidae species used?**

**-> Except for Anisakis simplex and Pseudoterranova decipiens, positive samples could not be obtained in Korea, so plasmid DNA was used.**

**The manuscript lacks electrophoresis of the products after the PCR reaction, and these products should be sequenced to finally confirm the specificity of the amplification.**

**-> The results of the sequence analysis are included according to Figure 6.**

**Moreover, the manuscript lacks information on statistical analysis, number of tests performed, number of positive and negative results, etc.**

**-> The testing methods involved investigating genetic analysis techniques for two types of parasites through literature review, focusing on real-time genetic testing methods as the subjects of analysis. To find the testing method with optimal sensitivity, each real-time genetic testing method was compared using one positive sample. Based on the results, the minimum detection limit, specificity, and reproducibility were analyzed.**

**Furthermore, as the authors emphasized at the beginning of the paper, there are currently verified methods for the detection of species from the Anisakidae family. This study does not introduce any new detection methods, and it is difficult to verify whether this probe is better than others currently available because the authors did not provide sufficient information and did not discuss the results based on other work.**

**-> The testing methods investigated the genetic analysis methods for two types of parasites through the paper, focusing on real-time genetic testing methods as the subjects of analysis. Additionally, confirmation was conducted using a new testing method.**

**To find the testing method with optimal sensitivity, each real-time genetic testing method was compared using positive samples, with one test conducted for each method. As a result, the minimum detection limit, specificity, and reproducibility were analyzed. The findings indicated that a method was identified that showed no cross-reactivity and could detect even at minimal concentrations.**

**At the same time, the authors write about identification in food, which has also not been verified. A matrix of infected and non-infected foods should be created and analyzed.**

**-> As mentioned earlier, we anticipated that it would be difficult to obtain samples from food, so we did not conduct a separate comparison between infected and non-infected foods.**

**Apart from the methodological part, the introduction is laconic and the sentences are repeated. Much of the information, for example about the development cycle, is incorrect. For example, "predated upon by fish... where the larvae mature into adults in the intestines" in line 65 is complete nonsense.**

**-> Line 64~These crustaceans are then predated upon by fish or squid, where the larvae mature into adults in the intestines**

**These crustaceans, as well as fish and squid, act as intermediate/paratenic hosts, where the larvae develop into adults in the intestines.**

**※ Comments:**

**The manuscript also contains some linguistic errors. In line 77, for example, I think it should read "infectious" and it reads infected, which gives the sentence a completely different meaning. The text should be edited by a native English speaker.**

**-> Line 76 : The most commonly infected species is the Anisakis genus, followed by Pseudoterranova decipiens, and rarely Contracaecum and Hysterothylacium [12]**

**The most commonly infectious species is the Anisakis genus, followed by Pseudoterranova decipiens, and rarely Contracaecum and Hysterothylacium**
