# Supplementary material for: Comparative study of Taqman-based qPCR assay for the detection of Anisakis simplex and Pseudoterranova decipiens
Source: PLoS One. 2025 Apr 25;20(4):e0320724. doi: 10.1371/journal.pone.0320724 (PMC12027023; doi:10.1371/journal.pone.0320724)
Supplement: Attachment — Submitted filename: Response to Reviewers_PONE-D-24-33370R1.docx [file pone.0320724.s006.docx]

**A point-to-point response to reviewers’ comments**

**PONE-D-24-33370R1**

**Comparative study of Taqman-based real-time PCR assay for the detection of *Anisakis simplex* and *Pseudoterranova decipiens***

We would like to sincerely thank you for taking the time to review our paper and provide valuable feedback. We improved our paper based on reviewers’ revision requests, attaching the revised version of our paper.

Below is our point-by-point response to each comment.

**[Journal Requirement]**

**Comment :**

**Please review your reference list to ensure that it is complete and correct. If you have cited papers that have been retracted, please include the rationale for doing so in the manuscript text, or remove these references and replace them with relevant current references. Any changes to the reference list should be mentioned in the rebuttal letter that accompanies your revised manuscript. If you need to cite a retracted article, indicate the article’s retracted status in the References list and also include a citation and full reference for the retraction notice.**

Response:

We reviewed the reference list again and found one duplicate ([3] and [29]), which we corrected and reflected in the revised manuscript. On line 74, [29] replaced it with [3] and removed the number [29] in the reference list. In addition, we have added 8 references to the list that refer to the Materials and Methods and the Results and Discussion sections.

**[Review Comments to the Author]**

**Reviewer #2**

**Thank you for taking my comments into account in the manuscript.**

**Please also change line 185 in the Results and discussion chapter to the correct accession number of the genomic sequences mtDNA (a. simplex KC810002) (P. decipiens OK338708)**

Response: We've changed the accession numbers at line 186-187 based on your suggestion.

line 186-187 **:** designed based on the sequences of *A. simplex* (KC810002.1) and *P. decipiens* (OK338708): one for *A. simplex* and five for *P. decipiens* (Table 2).

**Reviewer #3**

The manuscript has shown improvement since the first revision. However, it still requires significant enhancements to meet publication standards. Below are my comments and suggestions for further refinement:

**Results and Discussion: While the results section is improved, the manuscript would benefit from a more in-depth discussion. Expanding the discussion section will enhance the manuscript's robustness and make it more suitable for publication. Consider addressing broader implications, limitations, and potential future research directions.**

Response: We have revised the Results and Discussion and Conclusion sections to complement each other as much as possible. Please refer to line 275-290 and line 301-314.

line 275-290: Our study was aimed to analyze potential foodborne pathogens related to food consumption habits and climate change using qPCR methods. Accordingly, we aimed to investigate and compare genetic testing methods for parasites that may cause acute or chronic infections in food items, which are not frequently reported in South Korea but are a concern for the future. This was done by reviewing domestic and international research papers to identify the optimal genetic testing method.

Among other molecular biology techniques, qPCR has been applied as a rapid detection method to a variety of microorganisms, including bacteria, viruses, and parasites due to its speed, accuracy, and sensitivity [28–31].

In particular, it was believed that qPCR would yield faster results compared to conventional PCR methods, so this study was conducted by using this approach. Additionally, we considered the PCR composition methods utilized by the Korea Food and Drug Administration for genetic testing of parasites during foodborne illness investigations.

Based on this, it is expected that accurate, rapid, and cost-effective identification of anisakids found in foods, such as fish and seafood, will be possible. In addition, a method for detecting stable genes other than mtDNA sequences needs to be developed. Quantitative experiments should also be conducted based on qualitative experiments that determine the presence of parasites.

line 301-314: Anisakids are typically identified using traditional morphological dentification; however, achieving accurate genus identification is often challenging using the naked eye [9]. Therefore, a genetic approach with high accuracy is required to address this challenge effectively.

Recently, qPCR methods targeting various regions have been developed for the detection of Anisakis [2, 17, 22, 24–26, 32]. In this study, we designed our own primer/probe set targeting the cytochrome c oxidase subunit II (COX2) region to develop a more stable detection method in several mitochondrial genomes.

In summary, this study developed an effective qPCR detection method for *A. simplex* *and P. decipiens* that has the potential to be standardized. As a result of comparing genetic optimized methods for detecting two types of parasites, it was able to identify a method with high sensitivity and specificity in the laboratory. Consequently, the sequenced qPCR products of the selected primer/probe sets confirmed that each resulted in the target parasite by phylogenetic analysis. This method is expected to assist in the future identification of these two parasites using genetic amplification techniques.

**qPCR Section: It is essential for the authors to adhere to the Minimum Information for Publication of Quantitative Real-Time PCR Experiments (MIQE) guidelines (Bustin et al., 2009). Please ensure compliance with these guidelines and include a completed MIQE checklist in the supplementary data to strengthen the methodological transparency and reliability.**

Response: Based on your suggestions, we have organized the checklists applicable to our paper into a supplementary table (S1 table) and added them to the qPCR optimization section on lines 150-151.

line 150-151 : For developing the detection method, the MIQE guidelines was used to strengthen methodological approach in experiments. The checklist was summarized in the S1 table.

**Line 45: It is recommended that the nomenclature adheres to the MIQE guidelines. Specifically, the term "quantification cycle (Cq)" should be used for consistency and clarity.**

Response: line 45: sensitivity was checked by comparing the C_q_ values from the extracted DNA.

**Line 130: The term "Universal mater mix" should be corrected to "2X Universal Master Mix."**

Response: line 141: 10 μL of 2ⅹUniversal mater mix

**Lines 134 & 135: The term "PCR condition" would be more appropriately replaced with "thermocycle condition."**

Response: line 145: The thermocycle conditions, line 146: In-house thermocycle conditions

**Line 133: The term "PCR amplification" should be revised to "qPCR amplification" as the instrument used is a real-time PCR system.**

Response: line 144: qPCR amplification was performed using the 7500 Real-Time PCR System (Applied Biosystems, USA).

**Line 137: The nomenclature for "real-time PCR" should adhere to the MIQE guidelines. Specifically, use "qPCR" for quantitative real-time PCR and "RT-qPCR" for reverse transcription-qPCR. Please ensure this correction is applied consistently throughout the manuscript.**

Response: We have revised the wording (real-time PCR → qPCR) on whole manuscript with Table 4, Table 5 and the title based on your suggestions.

**Line 175: The phrase "thirteen detection methods" is confusing and could be misleading. It would be more appropriate to use "thirteen sets of primers/probes" for clarity and accuracy.**

Response: line 187: In total, thirteen sets of primers/probes were selected for the comparison between *A. simplex* and *P. decipiens* (Table 1, 2).

**Lines 181-183: The statement "According to Figure 6..." requires clarification regarding the algorithm used in the analysis. Was it Maximum Likelihood (ML), Neighbor-Joining (NJ), or Bayesian** **inference? Please specify the algorithm in the statement for better transparency and understanding.**

Response: Phylogenetic analyses were performed using Maximum Likelihood and are described in detail in the Materials and Methods – PCR product verification section. (Line 165-174)

line 165-174:

**PCR product verification**

Sanger sequencing was performed with the finalized primer/probe sets. The amplicons were subjected to PCR purification using the AccuPrep® PCR/Gel Purification Kit (Bioneer, Daejeon, Korea). This was followed by a cycling step with the BigDye Terminator kit v. 3.1 (Applied Biosystems, USA) and cleaned up with the BigDye XTerminator v. 3.1 (Applied Biosystems, USA). The cleaned products were sequenced by 3500 Genetic Analyzer (Applied Biosystems, USA).

Sequenced data were aligned to 9 nucleotide sequences which is registered in NCBI and phylogenetic analysis was performed MEGA 11, using the Maximum Likelihood method and Tamura-Nei model [35].

**[Reviewer #5]**

The authors have addressed a useful tool, but the manuscript is not well drafted.

Major concerns

**1. The authors should thoroughly review methods for nematode detection in introduction part, such as reverse PCR, LAMP, ddPCR. There must be successful methods in model nematode species.**

Response: We have added a further reference to the detection of Anisakidae family in the Introduction (line 91-94).

line 91-94: For the detection of Anisakidae larva, molecular biological methods such as restriction fragment length polymorphism (RFLP), recombinase polymerase amplification (RPA) and loop-mediated isothermal amplification (LAMP) assay have been investigated [15-20].

**2. The materials and methods part should be rephrased, such as primer design, sample collection and qPCR validation. The positive control is a part of sample collection. The authors should list all sample used in the manuscript, such as how and where the samples were collected. The authors should collect marine samples affected with nematode to validate the method. Inter-examinator validation belongs to technological repeats, which should be added to the part of qPCR. Besides, a melt curve should be carried out for each pair of primers.**

Response: First, based on your suggestions, the paragraph reorganization has been fixed. Materials and methods were divided into Specimen preparation and qPCR validation, and Positive control and Inter-examiner validation were separated into their own parts and placed as sub-sections.

Next, all *Anisakis simplex* *and Psuedoterranova decipiens* samples obtained from the Korea Institute of Cetacean Research Institute, National Institute of Fisheries Science, and other research institution in Korea. This is mentioned in the Materials and Methods - DNA extraction section.

Finally, in this study, the test method was developed using the Taqman probe method, and the PCR products were sequenced to identify the parasite species. Therefore, the reliability and accuracy of the detection method of primers/probes were confirmed by the sequencing results, and accordingly, the melt curve experiment was not conducted.

**3. Figures and tables are cited confusedly, which should be arranged in turn.**

Response: We realized that some of data was either not mentioned or incorrectly mentioned, so we've revised it.

line 216-220, 241-246, 294-298: The figures have been rearranged.

**4. The results part should be rephrased according to the materials and methods part in new revision.**

Response: The Materials and Methods sections have been reorganized as the suggested direction, but the Results and Discussion have been kept in their original order for flow of content. DNA extraction - primer/probe screening (sensitivity) - specificity - sequencing - Examiner validation is organized as follows.

**5. The conclusion part is too long. Some parts should be moved to discussion part.**

Response: We have revised the Results and Discussion and Conclusion sections to complement each other as much as possible. Please refer to line 276-291 and line 301-314.

line 275-290: Our study was aimed to analyze potential foodborne pathogens related to food consumption habits and climate change using qPCR methods. Accordingly, we aimed to investigate and compare genetic testing methods for parasites that may cause acute or chronic infections in food items, which are not frequently reported in South Korea but are a concern for the future. This was done by reviewing domestic and international research papers to identify the optimal genetic testing method.

Among other molecular biology techniques, qPCR has been applied as a rapid detection method to a variety of microorganisms, including bacteria, viruses, and parasites due to its speed, accuracy, and sensitivity [28–31].

In particular, it was believed that qPCR would yield faster results compared to conventional PCR methods, so this study was conducted by using this approach. Additionally, we considered the PCR composition methods utilized by the Korea Food and Drug Administration for genetic testing of parasites during foodborne illness investigations.

Based on this, it is expected that accurate, rapid, and cost-effective identification of anisakids found in foods, such as fish and seafood, will be possible. In addition, a method for detecting stable genes other than mtDNA sequences needs to be developed. Quantitative experiments should also be conducted based on qualitative experiments that determine the presence of parasites.

line 301-314: Anisakids are typically identified using traditional morphological dentification; however, achieving accurate genus identification is often challenging using the naked eye [9]. Therefore, a genetic approach with high accuracy is required to address this challenge effectively.

Recently, qPCR methods targeting various regions have been developed for the detection of Anisakis [2, 17, 22, 24–26, 32]. In this study, we designed our own primer/probe set targeting the cytochrome c oxidase subunit II (COX2) region to develop a more stable detection method in several mitochondrial genomes.

In summary, this study developed an effective qPCR detection method for *A. simplex* *and P. decipiens* that has the potential to be standardized. As a result of comparing genetic optimized methods for detecting two types of parasites, it was able to identify a method with high sensitivity and specificity in the laboratory. Consequently, the sequenced qPCR products of the selected primer/probe sets confirmed that each resulted in the target parasite by phylogenetic analysis. This method is expected to assist in the future identification of these two parasites using genetic amplification techniques.

**[Reviewer #6]**

The manuscript “Comparative study of Taqman-based real-time PCR assay for the detection of Anisakis simplex and Pseudoterranova decipiens” describes Taqman qPCR-based assay for the detection of two parasites. There are several points that the authors need to address:

**Language Issues:**

**1- The authors need to revise the language of the manuscript. There are instances of wrong word order and sentence structure.**

**a. Line 60-61: … infected raw fish infected**

**b. Line 67: comma is not needed here-… symptoms, such as…**

**c. Line 73: do not use semi-colon. Use full-stop - …reported; thus…**

**Methods:**

**Line 119-121: it is not clear which probe concentration is for which target.**

**Line 147: it seems a 5 has been missed here (10-5 -fold (1ⅹLOD) and 10-5 -fold 148 (5ⅹLOD))**

Response: Based on what you mentioned, we fixed all the language errors. Also, in line 141-143, we conducted experiments with cross-combinations of the 4 proposed concentrations, which are described in more detail in the Results section (line 207-215). Please refer to the Table 4 and Fig.4 for additional details.

In general:

**Since the authors prepared standards, they need to provide Limit of Quantification (LOQ). The LOS provided does not seem correct. The researchers need to first determine the LOQ and then determine LOD, based on a 2-fold serial dilution.**

Response: In this research, we aimed to find the optimal method for detecting acute or chronic infections caused by parasites in foods that are not frequently reported in Korea but are of concern for the future. We believe that real-time PCR methods would provide faster results than conventional PCR methods, which is why we chose this approach for our study. Therefore, the aim of this study was to develop a test method for the rapid and accurate detection of parasites in food, and only the LOD was performed, focusing on qualitative analysis. Further necessary experiments are mentioned in the Discussion.

**In the materials and methods section, no sensitivity study has been mentions. The authors need to point out the sensitivity study.**

Response: The sensitivity tests were screened with the mixture compositions and qPCR conditions presented in the Adjustment of qPCR methods section (line 138-151). The primer/probe sets summarized in Table 1 and 2 were used for each condition.

**Did the author try real samples? Did they use approve positive and negative samples?**

Response: We deemed it appropriate to use samples isolated from *Anisakis simplex* *and Psuedoterranova decipiens* obtained from the Korea Institute of Cetacean Research Institute, National Institute of Fisheries Science, another research institution in Korea. This is mentioned in the Materials and Methods - DNA extraction section.

**The author conducted inter-examiner study. However, they need to conduct inert-assay and intra-assay experiments.**

Response: For inert-assays, the stability has been tested with synthesized positive controls, which are presented at the beginning of the Results and Discussion section (line 190-192). For intra-assays, all experiments (sensitivity test, specificity test, etc.) were performed in duplicate and the data were organized as the average value. All data are within acceptable error limits.

line 190-192 : The positive control was reliably amplified within the expected range of Ct values in all experimental replicates. The Ct value was found to be 13.83 ± 0.01 for *A. simplex* and 13.84 ± 0.12 for *P. decipiens*, demonstrating the high reproducibility and consistency of the experiment.
